# Lysosomal activity in response to the incubation of pristine and functionalized carbon nanodots

**DOI:** 10.1016/j.isci.2024.111654

**Published:** 2024-12-25

**Authors:** Carla Sprengel, Céline David, Lena Berning, Cathrin Nollmann, Thomas Lenz, Kai Stühler, Björn Stork, Thomas Heinzel

**Affiliations:** 1 Solid State Physics Laboratory, Heinrich Heine University Düsseldorf, 40204 Düsseldorf, Germany; 2 Institute of Molecular Medicine I, Medical Faculty and University Hospital Düsseldorf, Heinrich Heine University, 40225 Düsseldorf, Germany; 3 Molecular Proteomics Laboratory, Biological Medical Research Center, Heinrich Heine University Düsseldorf, 40225 Düsseldorf, Germany; 4 Institute of Molecular Medicine I, Proteome Research, Medical Faculty and University Hospital Düsseldorf, Heinrich Heine University Düsseldorf, 40225 Düsseldorf, Germany

**Keywords:** Natural sciences, Chemistry, Applied sciences

## Abstract

We present functional studies of lysosomes in human cells after uptake of carbon nanodots (CNDs). Even under high CND concentrations, the lysosomal functionality, as characterized via cathepsins B and L as well as the autophagic markers SQSTM1/p62 and LC3B-II, is maintained. Furthermore, branched polyethylenimine (bPEI) molecules have been coupled to the CNDs as a model functionalization or example of a drug. We observe that the bPEI-CND conjugates accumulate to a higher degree in the lysosomes as compared to bPEI or CND alone. Here, changes in the lysosomal size and function are observed, which can be explained exclusively by the bPEI. It is concluded that CNDs are highly efficient and inert carriers for functional molecules into lysosomes as target, with the added value that lysosomal escape is suppressed, thereby avoiding unintended side effects in other cellular compartments.

## Highlights

• Carbon nanodots accumulate in lysosomes but do not influence the lysosomal activity
• Branched PEI is carried by the nanodots into lysosomes and remains active therein
• Branched PEI coating suppresses the escape of carbon nanodots from lysosomes
• The bPEI-nanodot conjugate can be regarded as a model drug delivery system

## Graphical abstract

**Figure:**
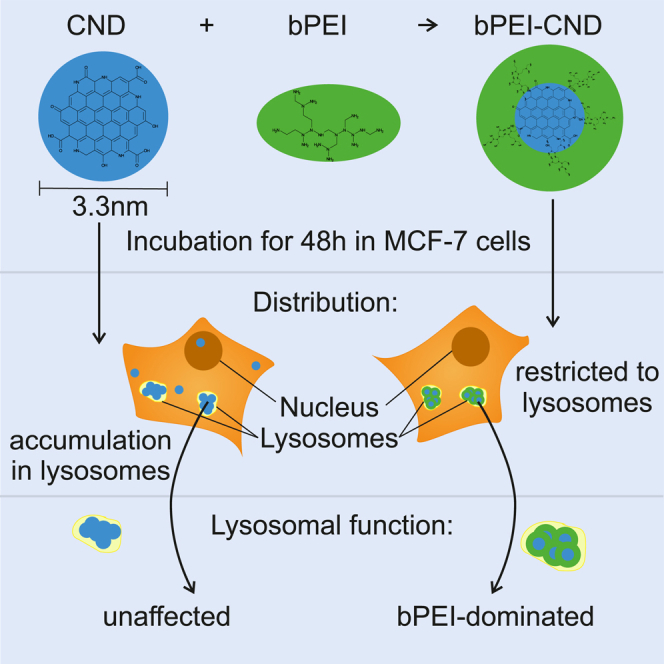

## Introduction

Carbon nanodots (CNDs) are promising fluorophores for cellular imaging and drug delivery and represent a stimulating alternative to conventional quantum dot systems, see ref.^1^ for a recent review. They are composed of one, or stacks of up to a few, graphene flakes. As compared to the more established II/VI-semiconductor based fluorescent quantum dots,^2^ they have a one to two orders of magnitude lower mass. Their high surface-to-volume ratio in combination with the hydrogen termination of the carbon atoms at the edge makes them well suited for functionalization.^3^ They show intrinsically a high solubility in water,^4^^,^^5^ while conventional quantum dots require a suitable coating. Furthermore, their low toxicity has been well established,^6^^,^^7^ in contrast to the II/VI quantum dot cores, which contain toxic metals such as Cd or In and require a protective layer, which adds to their complexity. On the other hand, the optical properties of many CND versions applied to cell experiments are poorly understood and have so far resisted attempts to design them toward a selectable frequency window. Therefore, CNDs complement the already established portfolio of available fluorescence markers. They appear particularly useful for applications where small, inert fluorophores are preferred.

In recent years, several studies have addressed the CND uptake by cells, their intracellular distribution as well and the metabolic and genetic response of the cells to their presence even at very high concentrations. Remarkable results have been reported, like for example, their suitability as intracellular pH sensors,^5^^,^^8^ application in photosensitizing experiments^9^^,^^10^ or the observation that the gene expression of the cells remains essentially unaffected in the presence of even approximately one hundred million CNDs per cell.^7^

This raises expectations regarding their application in drug delivery,^11^^,^^12^^,^^13^ with hopes that their presence neither alters drug effects nor causes side effects by themselves. For example, it has been reported that CNDs can improve the anti-cancer activity of cisplatin ^14^ and doxorubicin.^15^^,^^16^ In this context, the distribution pathway of the CNDs in the cell after exposure is relevant. It has been shown that pristine CNDs are primarily taken up via the endolysosomal pathway and end up at large concentrations in the lysosomes^17^ and to a lesser extent in the cytoplasm and in the nucleus, particularly in the nucleoli.^18^ Therefore, the question naturally arises as to whether the CNDs modify the physiology or metabolism of the lysosomes. Furthermore, when considering CND-mediated drug delivery, one could envisage protocols where the lysosome is the primary therapeutic target, such as enzyme replacement therapy, which has shown successes in the treatment of the quite severe lysosomal storage disorders, as being particularly promising.^19^ One related aspect of such applications is the development of protocols with the objective to increase the localization of the CNDs in the lysosomes.

In this context, it is relevant to clarify how CNDs as well as conjugates formed by CNDs and projectile molecules influence the lysosomal metabolism and trafficking, which comprises such different tasks as the degradation of proteins and extracellular particles, nutrient sensing or catabolite export.^20^ Furthermore, CNDs may be suitable as carriers for selective drug delivery into lysosomes, while possible carrier effects remain to be evaluated.

In the present study, we expose MCF-7 cells to pristine CNDs as well as to CNDs conjugated to branched polyethylenimine (bPEI) with a molecular weight of 600 Dalton (bPEI-CNDs) up to high concentrations, where the bPEI, a cationic polymer with abundant amine groups, plays the role of a test molecule to be delivered into the lysosome.^21^^,^^22^ The bPEI molecule was chosen because it is well established as a vehicle for non-viral gene or drug delivery,^23^ for example to deliver doxorubicin to the nucleus^24^ or to stabilize fragile proteins during transduction.^25^ The transfection via bPEI is based on its ability to buffer the pH by binding H^+^ ions, which can ultimately lead to rupture of the acidified endosomes or lysosomes, allowing the encapsulated particles to enter the cytosol.^21^^,^^26^ Furthermore, bPEI can be regarded as a drug by itself with functions such as gastric emptying,^27^ blockage of fibrin formation^28^ or the enhancement of the permeability of Gram negative bacterial membranes.^29^ These wide-spread applications enable conclusions regarding greatly varying aspects of the studied effects.

We characterize the functionality of the lysosomes via monitoring the expression levels or activity of lysosomal markers, such as the enzymes cathepsin B and L, or the autophagy-related markers SQSTM1/p62 and LC3B-II (LC3) in response to the exposures. The multifunctional protein p62 mediates the recruitment of damaged or foreign proteins to the autophagosomal pathway which leads to the lysosomal degradation of the proteins and the autophagy receptor p62 itself.^30^^,^^31^^,^^32^ Since p62 is degraded during this process, a decrease or increase in its level indicates enhanced or hampered autophagy, respectively. LC3 is another well-established marker for autophagy-related processes, since it is involved in autophagosome formation as well as the binding of p62-marked cargo.^33^^,^^34^ Due to its partial degradation during the digestion,^35^ LC3 levels can be used for sensing the autophagic activity as well. Cathepsins belong to the group of lysosomal hydrolases and are part of a variety of processes including the degradation of proteins.^36^^,^^37^^,^^38^ By monitoring their activity and the expression levels of the two autophagy markers, conclusions about the status of the lysosomes are drawn, and the effects of the bPEI bound to CNDs on the lysosomal function in comparison to both pristine CNDs as well as free bPEI are specified.

## Results and discussion

### Successful functionalization of carbon nanodots

We begin by characterizing the CNDs and functionalized bPEI-CNDs. Detailed physical and chemical characterization of the pristine CNDs was done by Fasbender et al.^7^ and their results are described briefly in the methods section later in discussion. Here, we therefore focus on the effects of the functionalization on the fluorescence and ^1^H-NMR spectra.

**Figure 1:**
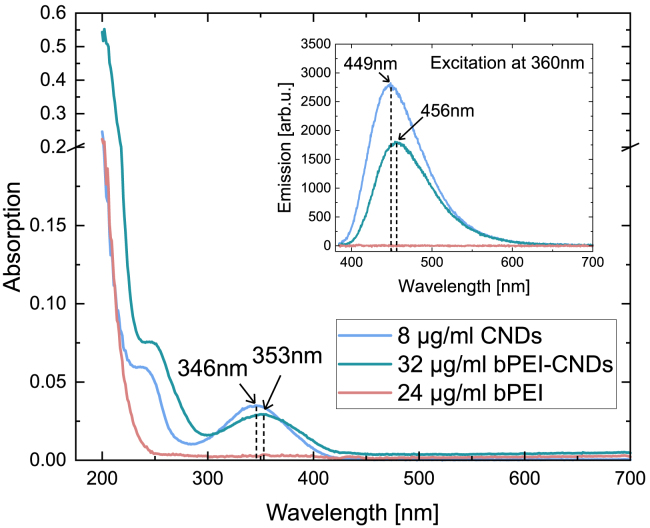
Absorption and emission spectra of pristine CNDs, bPEI and bPEI-CNDs Main figure: Pristine CNDs show an absorption maximum at 346 nm and a shoulder around 240 nm. The absorption of bPEI-CNDs is slightly red-shifted to 353 nm respective 245 nm. The inset shows the fluorescence spectra under excitation at 360 nm wavelength. CNDs show an emission maximum at 449 nm. The bPEI-CND emission is slightly red-shifted to 456 nm. Only the CNDs contribute to the fluorescence detected in this study.

The fluorescence and absorption spectra of the pristine CNDs, shown in Figure 1, are all in good agreement with findings reported elsewhere.^4^^,^^39^ They show an absorption peak at 346 nm and a shoulder in the deeper UV-region around 240 nm. After an excitation with 360 nm, the fluorescence signal with a maximum at 449 nm can be observed. Following the functionalization, the absorption and fluorescence spectra of the bPEI-CNDs are slightly red shifted. Their absorption peaks appear at 353 nm and 245 nm. The emission maximum is now located at 456 nm bPEI shows no absorption resonances and does not fluoresce. Furthermore, the functionalization of CNDs leads to a lower absorption at the same CND mass concentration compared to the pristine CNDs. An increase by a factor of 4 in the mass concentration of the bPEI-CND particles results in the same absorption at the maximum around 350 nm as the pristine CND absorption, indicating that a CND contributes ≈25% of the mass of a bPEI-CND conjugate. Moreover, the fluorescence spectra indicate that a covalent bond between the CNDs and bPEI has been established, most likely via unsaturated sites at the CND edges. We chose the absorption as a measure for the functionalization degree, because the CND fluorescence seems to be stronger influenced by functionalization. As displayed in Figure S1, the absorption of the CNDs is only slightly changed by the functionalization process with NHS and EDC, but the fluorescence emission (Figure S2) is weakened compared to the pristine CNDs. The modified and new surface groups of the CNDs may cause non-radiating electron relaxation.

**Figure 2:**
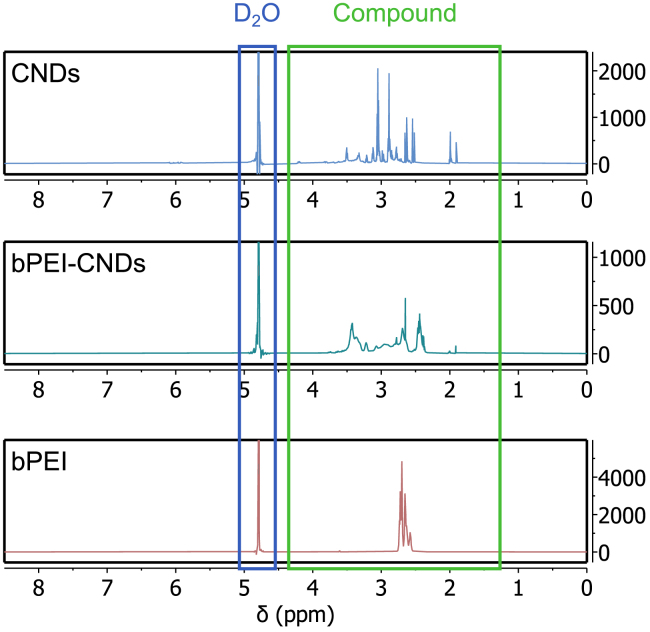
^1^H-NMR spectra of CNDs, bPEI-CNDs, and bPEI diluted in D_2_O The D_2_O peak of the solvent is visible at 4.79 ppm. bPEI-CNDs show broadened peaks compared to pristine CNDs, indicating a successful functionalization. See also Figures S3–S5.

As seen in Figure 2, the ^1^H-NMR spectrum of pristine CNDs shows narrow compound peaks between 1.99 ppm and 4.85 ppm (detailed figures in the supplement Figures S3–S5). After the functionalization, the CND peaks are broadened, indicating their successful bond to larger molecules (in this case bPEI). We tentatively explain this by a lower rotational diffusion due to an enlarged particle size. As a consequence, the direct nuclear spin-spin coupling, which is dependent on the angle between the external magnetic field and the binding vector between two atoms, is no longer averaged out, such that the peaks of the bPEI-CNDs appear broadened compared to those of pristine CNDs.

### Carbon nanodots and branched polyethylenimine-carbon nanodots accumulate within lysosomes

**Figure 3:**
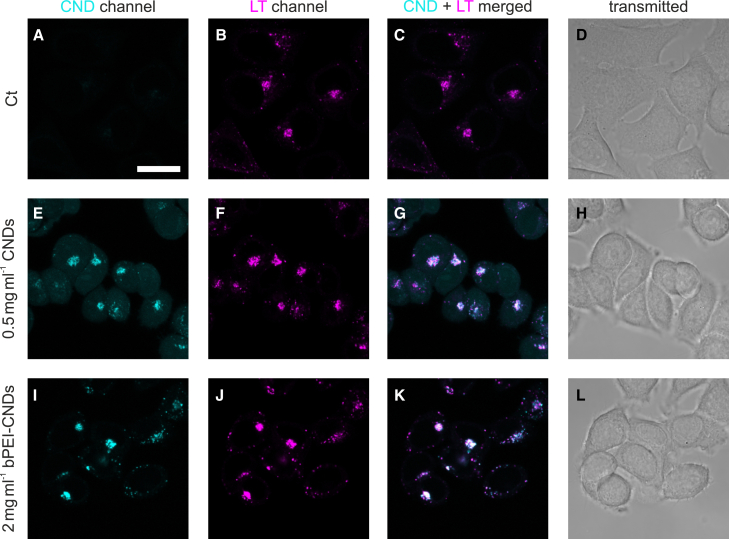
Confocal microscopy images of MCF-7 cells incubated with CNDs or bPEI-CNDs (A–D) MCF-7 cells without (bPEI-) CND incubation as a control. Lysosomes are stained with a lysotracker. The CND channel is shown in cyan, and the lysotracker (“LT”) channel is in magenta. The scale bar of 20 μm shown in A applies to all images. (E–H) MCF-7 cells incubated with 0.5 mg mL^−1^ CNDs. (I–L) MCF-7 cells incubated with 2 mg mL^−1^ bPEI-CNDs. Merging of the cyan CND channel and magenta LT channel results in blue-white color-signals. As seen in the CND-LT-overlay images G and K, CNDs and bPEI-CNDs mainly accumulate inside the lysosomes.

We begin the cellular experiments by characterizing the distribution of the CNDs and of the bPEI-CNDs within the cells. In Figure 3, microscopy images of the MCF-7 cells after CND - as well as after bPEI-CND - incubation are shown. For lysosomal staining, lysotracker was used. By merging the cyan (bPEI-)CND channel and the magenta lysotracker channel (Figures 3G and 3K), it emerges from the appearance of the mixed color that (bPEI-)CNDs accumulate mainly in the lysosomes, in accordance with earlier reports.^17^^,^^39^ Due to the imaging of living cells with moving organelles and a time delay between the measurement of the two fluorescence channels, a slight displacement of the overlaying CND and lysotracker signals may be observed. Additionally, Figure S6 shows representative intensity line plots of the microscopy images. The overlay of the cyan (bPEI-) CND peaks and magenta lysotracker peaks indicates the colocalization of CNDs and lysosomes. Moreover, the Pearson coefficients between 0.57 and 0.67 for all (bPEI-)CND samples (Table 1) indicate a strong colocalization of (bPEI-)CNDs and lysosomes.

**Table 1:** Pearson correlation coefficients determined between the (bPEI-)CND- and lysosomal lysotracker-channel of microscopy images of MCF-7 cells incubated with CNDs and bPEI-CNDs

Sample	ct	0.5 mg mL^−1^ CNDs	0.5 mg mL^−1^ bPEI-CNDs	2 mg mL^−1^ bPEI-CNDs
Pearson Correlation ± SD	0.21 ± 0.03	0.57 ± 0.07	0.61 ± 0.07	0.67 ± 0.12

Representative images for each sample are displayed in Figures 3 and S7. The given values are the mean Pearson correlation coefficients ± SD from *N* = 4 images per sample. The coefficients between 0.58 and 0.67 of the CND and bPEI-CND samples indicate a strong correlation between the (bPEI-)CND localization and lysosome positions. Since there were no (bPEI-)CNDs in the control “ct” sample, a lower coefficient of 0.21 results from background noise and autofluorescence signals of the cells.

We emphasize that a weak signal of the pristine CNDs is visible across the cell, possibly indicating a small rate of CND leakage from the endolysosomal pathway. In contrast, a background signal originating from bPEI-CNDs in other cell compartments besides lysosomes is not detectable. The underlying offset in the CND channel of the CND line plots compared to the bPEI-CND ones also support this finding. Apparently, the bPEI molecules localize the CNDs inside the lysosomes. We tentatively explain this by the enlarged particle size and/or by an altered charge of the bPEI-CNDs preventing lysosomal escape. Alternatively, the bPEI may buffer or change the lysosomal pH^40^ and thus its functionality. Nevertheless, the microscopy images confirm a successful transport of functionalized CNDs, i.e., the transport of the compound bPEI-CND into the lysosomes, and therefore support the potential application of CNDs as carriers for lysosomal-targeting therapeutics.

### In contrast to free branched polyethylenimine, carbon nanodots, and branched polyethylenimine-carbon nanodots do not affect the cell viability after 48 h of incubation

**Figure 4:**
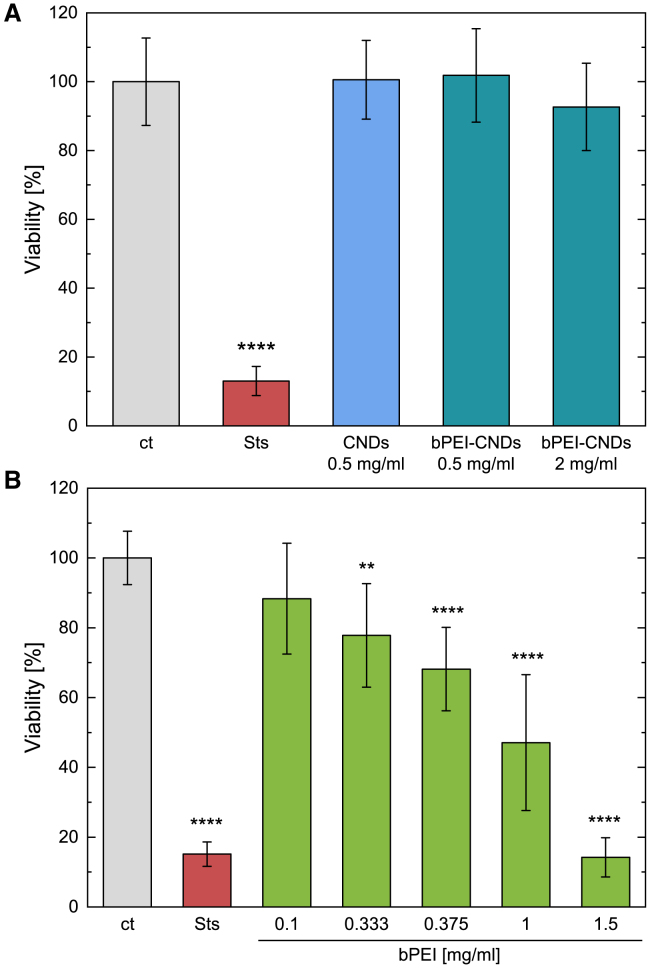
Viability of MCF-7 cells after incubation with CNDs and bPEI-CNDs or bPEI (A) Viability of cells after incubation with (bPEI-)CNDs. Staurosporine (Sts) was used as a positive control. Data are represented as mean ± SD of *N* = 3 biological replicates with each *n* = 3 technical replicates (expect bPEI 1 mg mL^−1^: *N* = 2, *n* = 3). *p* values were determined by one-way ANOVA with Bonferroni comparison. In comparison with the control sample “ct”: ∗∗*p* < 0.01, ∗∗∗∗*p* < 0.0001. (B) Liken A, for cells incubated with bPEI.

Since pristine bPEI does not fluoresce, only indirect conclusions about its intracellular distribution can be drawn from functional studies. The first of these studies comprises viability assays. Previous experiments have shown that an incubation with CNDs in a concentration of 0.5 mg mL^−1^ for 48 h does not alter the viability.^17^ We confirm this observation in our system and complement it by investigating the influence on the cell viability of the functionalized CNDs as well as the influence of unbound bPEI. As can be seen in Figure 4A, a concentration up to 2 mg mL^−1^ of bPEI-CNDs does not impact the viability significantly. Concentrations of free bPEI above 0.333 mg mL^−1^, however, do reduce the cell viability significantly, see Figure 4B. A free bPEI concentration of 0.375 mg mL^−1^ equals the concentration of the bound bPEI in the 0.5 mg mL^−1^ bPEI-CNDs solution and causes already a viability decrease to 68%. In addition, it is observed that a free bPEI concentration of 1.5 mg mL^−1^ which corresponds to a bound bPEI concentration in 2 mg mL^−1^ bPEI-CNDs leads to a viability of 14% which is similar to that one observed after incubation with the apoptosis-inducing and positive control staurosporine. The viability results of exposure of the cells to CNDs and bPEI-CNDs for 72 h and 96 h are displayed in Figure S8. CNDs do not influence the cells viability even after longterm exposure for 96 h. bPEI-CNDs on the other hand start to lower the viability significantly after 72 h when provided in high concentrations (2 mg mL^−1^) or after 96 h in lower concentrations (0.5 mg mL^−1^). After 96 h, the viability is significantly decreased to 80% or 70% after incubation with 0.5 mg mL^−1^ respective 2 mg mL^−1^ bPEI-CNDs.

We identify two possible reasons for this weakening effect of the CNDs on the bPEI-induced effects. First, the binding of bPEI to CNDs will lead to the capture of bPEI inside the lysosomes and thus to a reduction of cytotoxic effects of bPEI outside of the lysosomes. This is plausible considering the reported effects bPEI showed in other studies when entering via the lysosomal pathway.^41^ This explanation is in good accordance with the microscopy images (Figure 3) which show a strong localization of bPEI-CNDs inside the endo-lysosomal pathway. Second, possible cytotoxic effects of the bPEI inside the lysosomes may be attenuated due to the saturation of otherwise reactive bPEI sites by their bonds to the CNDs. We will revisit this explanation later in discussion.

### Carbon nanodots do not influence the cells’ proteome

To investigate the influence of the pristine CNDs on the cells’ proteome, MS-based quantitative differential (CND incubation vs. control) proteomics and secretomics analyses were performed using three (two for secretomics) different media (complete cell culture medium, basal medium RPMI1640 or starvation medium EBSS), resulting in a total of 3239 identified and quantified proteins. For the statistical analyses, the two values for relative protein quantification given by the MaxQuant software, the “intensities” and the “LFQ intensities,” were used (The “intensities” simply comprise the sum of all intensities of the identified peptides of a given protein in a given sample, while the “LFQ intensities” are calculated based on the intensities of the peptides that share their identification in the samples to be compared. Therefore, LFQ intensity values tend to be more accurate when there are enough shared peptides, but disregard many values from non-shared peptides, resulting in more missing data^42^).

**Figure 5:**
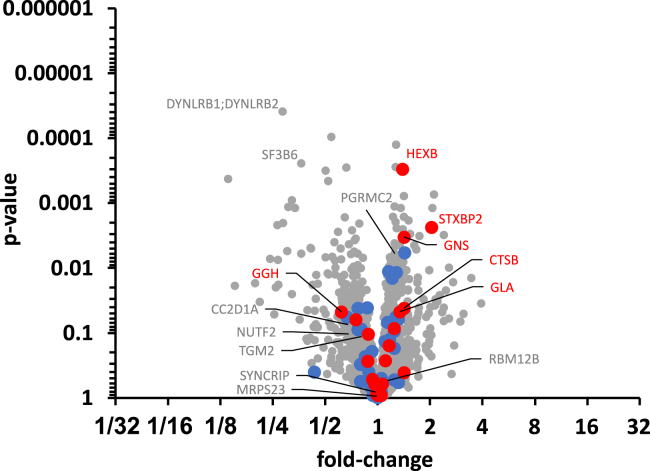
Volcano plot based on LFQ intensity values for MS-based proteomics Analysis of differential protein abundance (fold-change) after the CND treatment of MCF-7 cells in full culture media was performed of *n* = 5 replicates. See Figure S10 for the full set of proteome and secretome analyses in the three (two for secretomes) tested media. No proteins with a permutation-based FDR (SAM analysis) <5%  were identified in the present analysis. Gray gene name labels indicate proteins, for which this criterion was met for other conditions. Lysosomal lumenal and lysosomal membrane proteins are labeled with blue and red data points, respectively, and lumenal proteins with *p*-value <0.05  are labeled with red gene names.

Except for membrane-associated progesterone receptor component 2 (*PGRMC2*) in the proteome for the EBSS medium, no protein showed consistent significance (permuation-based FDR <5%, SAM analysis) for both the intensity- and LFQ intensity-based statistical analyses in neither full medium nor basal RPMI1640 medium and there was no protein that was significant over all analyses (see Figure 5 for an exemplary volcano plot; Figure S10 for the full set). Notably, none of the red or blue marked proteins associated with the lysosomal lumen and lysosomal membrane, respectively, were found to be significantly altered. Moreover, no profound functional connections or corresponding pathways were found for the significant proteins for the different conditions by a functional enrichment analysis using the STRING database^43^ (see Figure S11).

In the secretome data, no significantly altered proteins were detected (permuation-based FDR <5%, SAM analysis), indicating that pristine CNDs do not strongly alter the cellular secretion of proteins, and therefore also suggesting that pristine CNDs are inert to the metabolic processes of the cells.

### Carbon nanodots do not influence lysosomal functions and branched polyethylenimine-carbon nanodots show attenuated effects compared to free branched polyethylenimine

We proceed with functional studies of the lysosomes after exposure to the nanoparticles. The cellular pathway of autophagy represents a “recycling” mechanism that relies on functional lysosomes. Briefly, during autophagy, the cargo to be degraded is transported to lysosomes via double-membraned vesicles termed autophagosomes. Accordingly, monitoring the progress of autophagy using the marker proteins p62 and LC3 is an appropriate approach to analyze lysosomal activity. Similarly, lysosomal functionality can be assessed by monitoring cathepsin activity. Cathepsins are lysosome-resident hydrolases that ultimately contribute to the degradation of proteins. Specifically, we monitor the activities of cathepsin B and L, respectively. Here, we keep the compound concentrations in the regime where their effect on the viability is not significant or the viability of the cells is still above 75%.

**Figure 6:**
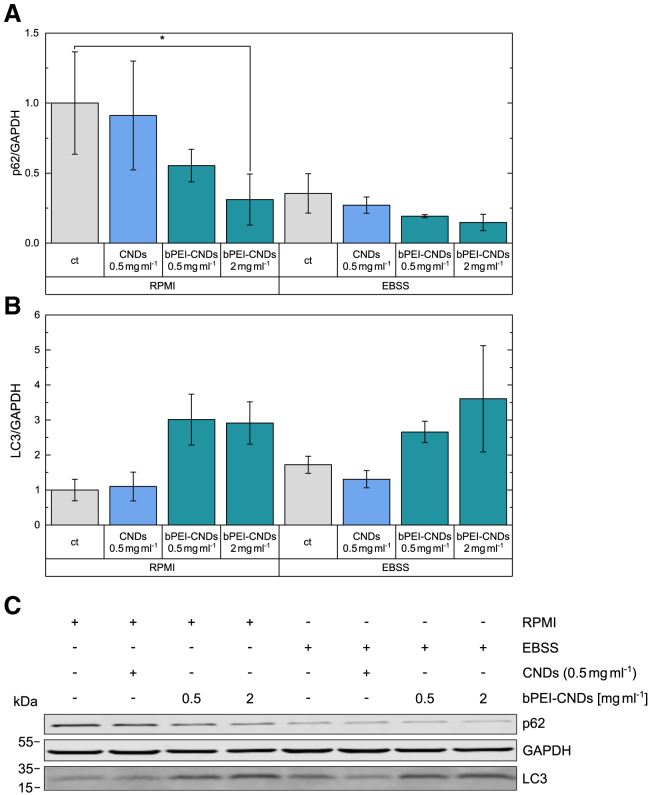
Influence of CNDs and bPEI-CNDs on cellular p62 and LC3 levels (A and B) Quantification of cellular (A) p62 and (B) LC3 levels. Samples were measured in biological triplicates. The levels were quantified and normalized to GAPDH. Data are represented as mean ± SD. *p* values were determined by two-way ANOVA with Bonferroni comparison. Shown is the significance compared to the control sample (“ct”) in the same medium: ∗*p* < 0.05. (C) Representative example of an immunoblot.

In the full cell culture medium (“RPMI”) containing serum and amino acids, we do not observe any significant effect of CNDs on p62 and LC3 levels, see the left hand side in Figure 6A. At high bPEI-CND concentrations (2 mg mL^−1^) the bPEI-CNDs lead to a significant decrease of the p62 level to 31% of the control, and a tendency toward decreasing p62 concentrations is already noticeable for the concentration of (0.5 mg mL^−1^). The LC3 levels are not significantly altered, nevertheless a tendency to increase LC3 levels can be seen in Figure 6B for bPEI-CND treatments. Induction of autophagy by starvation (right hand side, “EBSS”) reduces p62 levels, and this effect is not significantly altered by any additional treatment. Again, increased LC3 levels can be observed for bPEI-CND treatments. Collectively, CNDs do not appear to affect cellular autophagic capacity. In order to further characterize the observed effects for bPEI-CNDs, we combined bPEI (unbound) with bafilomycin A_1_. This compound is an inhibitor of the vacuolar H^+^-ATPase and ultimately inhibits the fusion of autophagosomes with lysosomes.^44^ The combination of a stimulus +/− bafilomycin A_1_ allows the analysis of the autophagic flux. As shown in Figure S12, bafilomycin A_1_ cannot prevent the bPEI-induced reduction of p62, indicating that autophagy-unrelated mechanisms contribute to this effect. Since the effects of unbound bPEI and bPEI-CNDs on p62 levels are similar, we assume that these autophagy-unrelated mechanisms also cause the effects on p62 and LC3 levels shown in Figure 6. Note that for unbound bPEI a reduced concentration by a factor of 4.5 compared to bound bPEI is needed to the effect the cell metabolism significantly.

**Figure 7:**
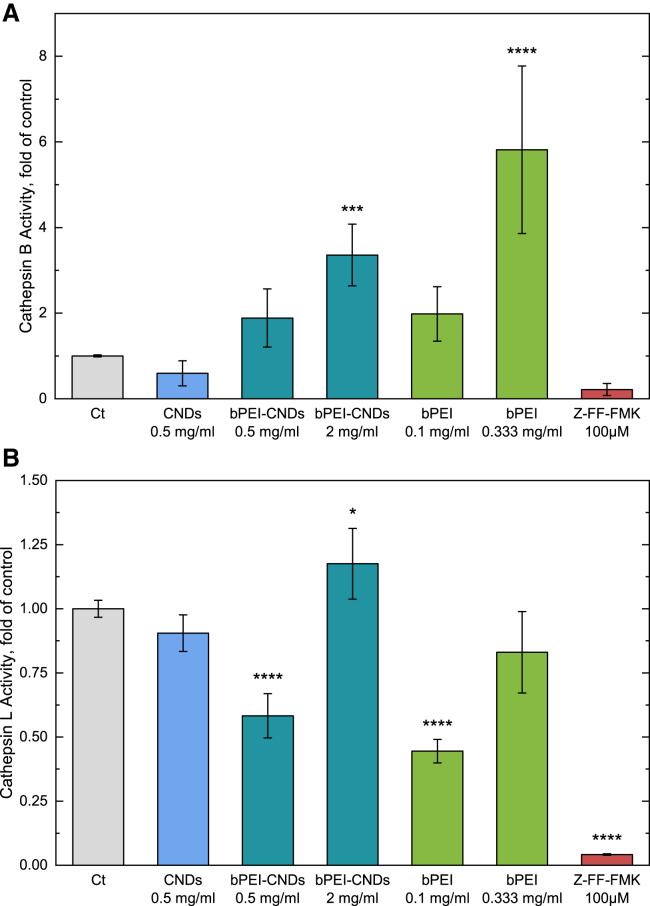
Activity of cathepsins after incubation with CNDs, bPEI-CNDs, and bPEI (A) The cathepsin B activity is displayed. The cathepsin inhibitor Z-FF-FMK was used as a positive control. The samples were measured in biological triplicates with technical duplicates each. Data are represented as mean ± SD. *p* values were determined by one-way ANOVA with Bonferroni comparison. In comparison with the control sample “ct”: ∗*p* < 0.05, ∗∗∗*p* < 0.001, ∗∗∗∗*p* < 0.0001. (B) Like A, for the Cathepsin L activity.

The activity of cathepsins B and L is not significantly altered upon CND incubation as seen in Figure 7, which supports the suitability of CNDs as inert carriers for drug delivery systems. After incubation with high bPEI-CND concentrations (2 mg mL^−1^), cellular cathepsin B activity is significantly enhanced by a factor of 3.3. Incubation with 0.333 mg mL^−1^ bPEI leads to a significantly increased cathepsin B activity by a factor of 5.8. Therefore bPEI incubation results in a higher cathepsin B activity regardless of the state (bound/unbound). Note that the effect of unbound bPEI on the cells' cathepsin B activity is attenuated upon CND binding. A concentration of 0.333 mg mL^−1^ unbound bPEI leads to a higher cathepsin B activity than a concentration of 2 mg mL^−1^ bPEI-CNDs which corresponds to approximately 1.5 mg mL^−1^ bound bPEI. The increased cathepsin B activities due to bPEI are in good accordance with a significantly increased level of cathepsin B inside the cells after free-bPEI incubation as shown in the immunoblots in Figure S13. This could be due to a general higher activity inside the lysosomes, as well as a larger total quantity due to a higher lysosomal number needed because of bPEI-mediated effects on the whole cell. Compared to the cathepsin B activity, for the cathepsin L activity, a concentration dependent effect of bPEI is observed. Lower concentrations of bPEI (bound in 0.5 mg mL^−1^ bPEI-CNDs or unbound 0.1 mg mL^−1^) lead to a significant decrease of the relative activity to 58% respective 44%. Upon bPEI concentration increase (e.g., bound in 2 mg mL^−1^ bPEI-CNDs or unbound 0.333 mg mL^−1^), the cathepsin L activity is significantly enhanced to 125% or back at an unaltered level. We assume that two different effects occur depending on the bPEI concentration, especially on the free reaction sites of bPEI. At low bPEI concentrations, a potential change of the pH value inside the lysosomes toward neutral could be a reason for the decreased cathepsin L activity as it was seen that bPEI can prevent the strong acidification of lysosomes.^40^ Due to the cathepsin L activity optimum in the acidic environment, at higher pH their activity is expected to be reduced. This is in good accordance with the unaltered cathepsin B activity at low bPEI concentrations since cathepsins B are known to show still good activity at less acidic environments.^45^ Note that the change of the lysosomal pH upon bPEI incubation is still under debate,^46^ such that other lysosome-affecting effects such as direct interactions with unsaturated bPEI-sites need to be considered as well. Since the cathepsin L activity is increased again to a normal or higher level at higher bPEI concentrations, a second bPEI-mediated effect should be considered. Taking the increased cathepsin B and L activity together, we suggest that the increased bPEI levels lead to cellular stress reactions, resulting in higher lysosomal activity. This is in good accordance with the immunoblotting and viability results which indicate autophagy-unrelated mechanisms as described above.

### Lysosomal size and abundance does not change upon carbon nanodot incubation but are influenced by free branched polyethylenimine and branched polyethylenimine-carbon nanodots

**Figure 8:**
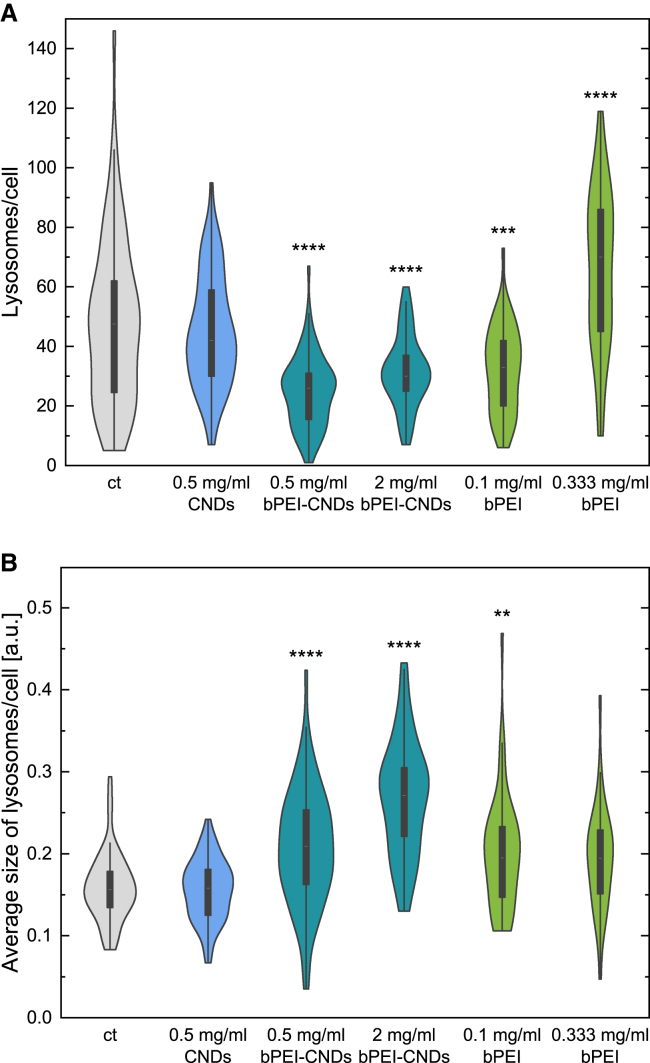
Violin plots of lysosome parameters as quantified from the microscopy data (A) Quantification of the number of lysosomes per cell. 34 to 72 cells per condition were analyzed. The accompanying boxplot shows the interquartile range, and the white line inside denotes the median. *p* values were determined by one-way ANOVA with Bonferroni comparison. In comparison with the control sample “ct”: ∗∗*p* < 0.01, ∗∗∗*p* < 0.001, ∗∗∗∗*p* < 0.0001. (B) Like A, for the quantification of the average size of lysosomes per cell.

Finally, we look at the size distributions of the lysosomes after incubation, see Figure 8. In agreement with all other observations, there is again no effect of the pristine CNDs on the number (Figure 8A) and the size (Figure 8B) of the lysosomes. Incubation of bPEI-CNDs leads to significantly fewer lysosomes per cell with larger average sizes. For pristine bPEI, concentration dependent effects are observed. Free bPEI causes the lysosomes’ size to increase and the number of lysosomes to decrease significantly at intermediate concentrations, similar to the effects of bPEI-CNDs. For large bPEI concentrations, the lysosomal size decreases again to a not significantly altered level compared to the control while the average number of lysosomes increases significantly compared to the control. Note that much smaller concentrations are needed for free bPEI (here 0.1 mg mL^−1^) to qualitatively reach the same effect as bPEI-CNDs. The effect of bPEI is, as seen in the other experiments as well, attenuated due to the binding to CNDs. An increased lysosomal size upon free bPEI or bPEI-CND incubation might be explained by an early-state osmotic swelling as a consequence of the proton buffering capacitance (“proton sponge effect”) of bPEI.^47^ Rupture of Lysosomes, usually observed when bPEI is used for transfection,^48^ is unlikely due to missing signal distribution of bPEI-CNDs across the whole cell as seen in the microscopy images (Figure 3). If other effects such as an enhanced lysophagy because of bPEI-damaged lysosomes occur needs to be further investigated. A different lysosomal positioning in the cell could also be the reason for the altered values. Since the lysosomal sizes are in the scale of the microscopic resolution, for lysosomes that cluster perinuclear, as seen upon lysosomal perturbations,^49^ the microscopic distinction could be difficult. This can lead to distorted values since multiple smaller lysosomes could be detected as one. For higher free bPEI concentration, the increased number of lysosomes and unaltered size compared to the control could be assigned to a general stress reaction of the cell against bPEI. This is also consistent with the previously discussed elevated cathepsin B activity and abundance, as well as slightly increased LC3 levels upon incubation with higher free bPEI concentrations, all suggesting the formation of new lysosomes.

### Conclusion

We investigated the effects of pristine fluorescent carbon nanodots on the cells’ proteome and on general aspects of the lysosomal activity. We found that even though high numbers of CNDs accumulate inside the lysosomes, they did not affect the lysosomal function nor the overall proteome. We furthermore examined the functionalizability of those CNDs by binding a relevant polymer (bPEI) to them and testing whether the CNDs can transport those compounds into the lysosomes as a target. We have seen indeed that the functionalized CNDs show a more correlated uptake into the lysosomes compared to the pristine CNDs. The results suggest that the binding to CND localizes the bPEI more targeted into lysosomes compared to the distribution of free bPEI inside the cells, resulting in better cell viability and probably less unwanted damage to other cell compartments. Moreover, we saw that the bound bPEI could still interfere the lysosomal metabolism as investigated by the autophagy markers LC3 and p62, the cathepsin B and L activity as well as seen by the influenced lysosomal size and quantity. We conclude that the CNDs are suitable as inert carriers in drug delivery systems and can transport the compound to be delivered precisely into the (endo-)lysosomal compartments without interfering with the overall viability of the cells. This could be particularly relevant for the treatment of diseases where the cell should remain intact, such as lysosomal storage diseases or neuro-degenerative diseases, but lysosomal functions should be altered. In such protocols, a possible attenuation of the therapeutic effects due to the binding of the compound to the CND needs to be assessed.

### Limitations of the study

All studies reported above have been observed on one cell line. However, since in earlier studies, the uptake of CNDs by live cells has shown only marginal cell-type specific variations, we expect that the behavior will be similar in other cell lines. It remains to be seen in future studies to what extent these lysosomal effects depend on the type/nature of the functional molecule attached to the CND.

## Resource availability

### Lead contact

Requests for further information and resources should be directed to and will be fulfilled by the lead contact, Thomas Heinzel (thomas.heinzel@hhu.de).

### Materials availability

This study did not generate new unique reagents.

### Data and code availability

All data (except MS proteomics and secretomics) are deposited at Mendeley Data: https://doi.org/10.17632/zgv3gpshpv.1 and are publicly available as of the date of publication. The mass spectrometry proteomics and secretomics data have been deposited to the ProteomeXchange Consortium via the PRIDE^50^ partner repository, PRIDE: PXD053105.

## Acknowledgments

We acknowledge support of L. Fastabend regarding the preparation of bPEI-CND compounds, as well as the use of microscope facilities at the Center of Advanced Imaging (CAI) at HHU and the CeMSA@HHU (Center for Molecular and Structural Analytics at the Heinrich Heine University) for measuring the NMR data. We Thank H. Neubauer for providing the MCF-7 cells used in this study. We acknowledge the support of the Institut für Physikalische Biologie at HHU for the lyophilization of the CNDs. T.H. and C.S. appreciate stimulating discussions with R. Haas. C.S. acknowledges financial support from the 10.13039/100010127Jürgen Manchot Foundation (no funding number).

## Author contributions

C.S., C.D., L.B., C.N., T.H., and B.S. conceived the experiments and analyzed and discussed the data. C.S. prepared and characterized the carbon nanodots and the conjugates, prepared the samples for the cell experiments, carried out the assays, some confocal microscopy measurements, the image analysis as well as the statistical analyses, and figure preparation. C.D. and L.B. performed the immunoblot analyses, and the cathepsin assays and contributed to the viability assays. C.N. assisted in the carbon nanodot functionalization and sample preparation for cell experiments as well as performed some confocal microscopy measurements. T.L. and K.S. developed the MS methodology. K.S. performed funding, supervision, and provision of MS infrastructure. T.L. performed MS investigation and sample workup, MS data analysis and curation, and interpretation and visualization of MS data. C.S. and T.H. wrote the first draft of the article. C.D., L.B., C.N., T.L., and B.S. contributed to the writing, reviewing, and editing of the final article. B.S. and T.H. supervised the project.

## Declaration of interests

The authors declare no competing interests.

## STAR★Methods

### Key resources table

**Table:** 

REAGENT or RESOURCE	SOURCE	IDENTIFIER
**Antibodies**		

Rabbit Polyclonal Anti-LC3B	CST	Cat#2775; RRID:AB_915950
Guinea pig Polyclonal Anti-p62/SQSTM1	PROGEN	Cat#GP62-C; RRID:AB_2687531
Rabbit Monoclonal Anti-Cathepsin B	CST	Cat#31718; RRID:AB_2687580
Mouse Monoclonal Anti-GAPDH	abcam	Cat#ab8245; RRID:AB_2107448
Mouse Monoklonal Anti-β-Actin	Sigma-Aldrich	Cat#A5316; RRID:AB_476743
IRDye® 680RD Donkey anti-Guinea Pig IgG Secondary Antibody	LI-COR Bioscience	Cat#926-68077; RRID:AB_10956079
IRDye® 800CW Goat anti-Rabbit IgG Secondary Antibody	LI-COR Bioscience	Cat# 926-32211; RRID:AB_621843
IRDye® 800CW Goat anti-Mouse IgG Secondary Antibody	LI-COR Bioscience	Cat# 926-32210; RRID:AB_621842

**Chemicals, peptides, and recombinant proteins**		

Anhydrous citric acid	Thermo Fisher	Cat#36664.22
DETA, Diethylenetriamine	Merck	Cat#8032740100
EDC, N-(3-Dimethylaminopropyl)-N′-ethylcarbodiimide hydrochloride	Merck	Cat#E6383
NHS, N-Hydroxysuccinimide	Merck	Cat#130672
Lysotracker™Deep Red	Thermo Fisher	Cat#L12492
MTT, Thiazolyl blue, 3-(4,5-Dimethylthiazol-2-yl-2,5-diphenyl-2H-tetrazolium bromide	Roth	Cat#4022
Sts, Staurosporine	Biozol	Cat#LCL-S-9300
Bafilomycin A_1_	Sigma-Aldrich	Cat#B1793
Z-FF-FMK, Cathepsin Inhibitor	Merck	Cat# 219421

**Critical commercial assays**		

Cathepsin B Activity Assay Kit	abcam	Cat#ab65300
Cathepsin L Activity Assay Kit	abcam	Cat#ab65306

**Deposited data**		

human sequence database	UniProtKB	Downloaded 01/27/2021
Mass spectrometry data	This paper	PRIDE: PXD053105
Data from the manuscript (except MS data)	Mendeley Data	Mendeley Data: https://doi.org/10.17632/zgv3gpshpv.1

**Experimental models: Cell lines**		

MCF-7 (female)	Hans Neubauer Lab, UKD Düsseldorf	N/A

**Software and algorithms**		

MestReNova	Mestrelab Research	14.2.0-26256 https://mestrelab.com/download
Omero.figure	OME	V6.0.1
Fiji/ImageJ with Plugin: BioVoxxel	Schindelin et al.^50^	V1.54f https://imagej.net/downloads
JACoP Fiji Plugin	Bolte and Cordelières^51^	https://imagej.net/plugins/jacop
Image Studio	LI-COR Bioscience	https://www.licor.com/bio/image-studio/
MaxQuant	Max Planck Institute for Biochemistry, Planegg, Germany	2.1.3.0 https://www.maxquant.org/
Siggenes Package in R	Bioconductor	https://www.bioconductor.org/packages/release/bioc/html/siggenes.html
OriginPro	OriginLab Corporation	2021b https://www.originlab.com/

**Other**		

Microwave	CEM	Discover
Dialysis devices, Float-A-Lyzer 0.1-0.5kDa and 3.5-5kDa	Repligen	#G235061 and #G235065
Microscopy slides, 8 well μ-Slide, ibiTreat	Ibidi	#80806

### Experimental model and study participant details

#### Cell culture

For all experiments, human female MCF-7 cells were used. The cells were cultured in RPMI 1640 (Biowest, #L0501) with 10 % Fetal Bovine Serum (FBS, Sigma-Aldrich, #F2442), 100 U ml^−1^ penicillin and 100 μg ml^−1^ streptomycin (Sigma-Aldrich, #P0781) and 300 mg l^−1^ L-Glutamin (Sigma-Aldrich, #G7513) in an incubator with humidified air at 37°C and 5% CO_2_. The cells were passaged every three to five days using Trypsin-EDTA solution (Sigma-Aldrich #T3924).

### Method details

#### Preparation of CNDs and bPEI-CND compounds

The CNDs were synthesized according to a modified version of the protocol of Qu et al.^4^ which has been described in detail elsewhere.^39^ Briefly, 210 mg anhydrous citric acid (Thermo Fisher, #036664.22) and 340 mg Diethylenetriamine (DETA, Sigma Aldrich, #8032740100) are stirred for 5 min at RT before the solution is heated in a sealed, teflon-lined microwave vessel under continuous stirring at 180°C for 2:30 min in a scientific microwave (CEM Discover). The product was dissolved in 10 ml deionized (DI) water, transferred to a dialysis device (Repligen, Float-A-Lyzer, 0.1-0.5kD, #G235061) and dialysed against 2 l DI water for 48 h with three water exchanges. After dialysis, the product was lyophilized to determine the final mass and dissolved with the targeted concentration in the required solvents for further experiments.

The functionalization of the CNDs was implemented with N-(3-Dimethylaminopropyl)-N′-ethylcarbodiimide hydrochloride (EDC, Sigma Aldrich, #E6383) and N-Hydroxysuccinimide (NHS, Sigma Aldrich, #130672) coupling. The lyophilized CNDs were solved in DI water in a concentration of 20 mg ml^−1^. After solving 1 g of EDC in 5 ml DI water, 10 ml of the CND solution were added and stirred for 10 min. 1 g of NHS was added and stirred for another 10 min before 200 mg of bPEI (Merck, #408719) was added. The final solution was stirred for 24 h. Afterwards the solution was transferred to dialysis devices (Repligen, Float-A-Lyzer, 3.5-5kD, #G235065) and dialyzed until the peak of the residual coupling reagents in the ^1^H-NMR spectrum (around 2.65 ppm) was sufficiently low (dialysis for at least 132 h with two water exchanges per 24 h). The final product was lyophilized to determine the dry mass and used to prepare the solutions for the experiments.

#### Characterization of the CNDs

Characterization of the unfunctionalized CNDs has been previously described by Fasbender et al.^7^ and led to the following results. CHN chemical elemental analysis revealed that the CNDs consist of 40% C, 19% N and 8% H atoms. The remaining fraction could be assigned to oxygen atoms by X-ray photoelectron spectroscopy (XPS) (see Figure 1A in ref.^7^, as well as Figure S1 in its Supplement). This method also revealed that 29% of the carbon bonds consist of C-C single bonds, while the remaining bonds were equally assigned to C-O and C-N bonds (Figure 1B in ref.^7^). Via Raman spectroscopy, it was shown that the C-C bonds consist of sp^2^- and sp^3^-hybridized carbon atoms, see Figure 1C in ref.^7^ Furthermore, COOH/C-OH and C=O/C-O edge functional groups were identified, and the D and G band signal of graphene at 1375*cm*^−1^ and $1596cm^{-1}$ can be seen in Figure S2 in the Supplement of ref.^7^ The mean particle size was measured via HRTEM and determined to be 3.3 nm with a FWHM of 0.6 nm, as shown in Figure 1C in ref.^7^ (the size distribution histogram is shown in Figure S3 of its Supplement). Atomic force microscopy studies show CND heights between 1 nm and 2 nm, indicative of up to three graphene layers per CND, see Figure S4 of ref.^7^ The fluorescence spectra revealed absorption peaks at 240 nm and 350 nm which can be assigned to *π*-*π*^∗^ transitions of C=C bonds and to *n*-*π*^∗^ transitions of C=O bonds, respectively.^4^ Excitation between 320 nm and 400 nm leads to a fluorescence emission with a maximum around 460 nm. The quantum yield for excitation at 360 nm was found to be 23%. These results have been reported in Figure 2 of ref.^7^

In this work, CNDs and bPEI-CNDs were synthesized as described above and their fluorescence and ^1^H-NMR spectra were used for characterization. The fluorescence spectra of CNDs, bPEI-CNDs and bPEI in DI water, shown in Figure 1, were obtained using a Horiba Duetta™ Fluorescence and Absorbance Spectrometer. Emission spectra were measured with excitation at 360 nm wavelength. For an independent confirmation of the successful functionalization, ^1^H-NMR spectra (600 MHz) of CNDs, bPEI-CNDs and bPEI in D_2_ O were recorded with a Bruker Avance III - 600 by the CeMSA@HHU. The data was then processed and displayed with MestReNova (14.2.0-26256).

#### Incubation with CNDs, bPEI-CNDs and bPEI

CNDs, bPEI-CNDs and bPEI were dissolved in DPBS (Dulbecco’s phosphate-buffered saline, Gibco, #14190144) and sterile filtered through a 0.2 μm PES membrane (Sarstedt, 83.1826.001) prior to their incubation with cells. The compounds were added to the culture medium, such that the final concentrations are obtained. All samples (including the control) were adjusted to contain the same amount of DPBS while retaining the correct compound concentration. The cells are then incubated for 48 h at humidified air with 5 % CO_2_ and 37°C. In the Table labeled “Compound concentration”, the selected compound concentrations are listed along with the corresponding justifications.

**Table:** Compound concentrations

Compound	Concentration[mg ml^−1^]	Justification
CNDs	0.5	no influence on cell viability after 48 h incubation^17^
bPEI-CNDs	0.5	mass-concentration equal to 0.5 mg ml^−1^ CNDs
bPEI-CNDs	2	particle concentration equal to 0.5 mg ml^−1^ CNDs
bPEI	0.1	no influence on cell viability after incubation for 48 h
bPEI	0.333	cell viability is above 75% after incubation for 48 h as seen in Figure 4
bPEI	0.375	equal to concentration of bound bPEI in 0.5 mg ml^−1^ bPEI-CND
bPEI	1.5	equal to concentration of bound bPEI in 2 mg ml^−1^ bPEI-CND

#### Confocal fluorescence microscopy

For confocal fluorescence microscopy with Lysotracker™ Deep Red (“lysotracker”, Thermo Fisher, Invitrogen™, #L12492) staining, the cells were seeded in a 8 well μ-Slide (ibidi, ibiTreat #80806) and treated for 48 h as described above. The basal culture medium was exchanged to phenol red-free RPMI 1640 (Biowest, #L0505). After the incubation time of 48 h, the medium was removed and new medium containing 50 nM lysotracker was added. After an incubation for 45 min in the dark at humidified air with 5 % CO_2_ at 37°C the medium was removed and fresh medium without lysotracker was added. The samples were directly imaged using a Zeiss LSM 710 with a Plan-Apochromat 63x/1,4 Oil objective and a closed sample chamber heated to 37 °C. The CNDs were excited with a 405 nm laser diode and the fluorescence was detected in the range of 410-580 nm (“CND channel”). The lysotracker was excited with a 633 nm HeNe Laser and the emission was detected in the range of 647-754 nm (“lysotracker channel”). The transmitted light images were obtained through the lysotracker channel. All images were obtained using the same measurement parameters. The images were plotted using OMERO.figure (v6.0.1).

#### Image analysis

To determine the amount and size of lysosomes per cell in each sample, the microscopy images were analyzed using Fiji^51^ (ImageJ v1.54f). First, background noise is removed through a convoluted background subtraction (median, radius:10) by the implemented *BioVoxxel* plugin and an intensity threshold is set. Afterwards, the amount and size of the lysosomes is determined using the *Analyze Particles* function (size: 0.01 μm^2^-Infinity, circularity: 0.00-1.00) provided within ImageJ. The macro used is provided in the supplement. For calculating the intensity lineplots, *Multichannel Plot Profile* in Fiji was used. Pearson Correlation Coefficients were determined with the *JACoP*^52^ Plugin in Fiji.

#### Viability assay

The cell viability was measured using an MTT assay (Roth, #4022). The cells were seeded in a 96-well plate in triplicates and incubated as described above for 48/72/96 h. For the positive control, cells were treated with 5 μM staurosporine (Sts, Biozol, #LCL-S-9300) for 48/72/96 h. Afterwards, 20 μl MTT solution (5 mg ml^−1^ in DI water) was added to each well and incubated for 40 min. The medium containing residual MTT was removed and 100 μl DMSO were added to each well. The plates were shaken gently until violet formazan was dissolved. The absorbance was measured at 570 nm and 650 nm (for reference) with a microplate reader (SynergyMx, BioTek, Winooski, VT, USA). The reference absorption at 650 nm was subtracted from the absorption at 570 nm. Afterwards the offset of wells without cells was set to zero. The mean value of the control cells was set to a viability of 100%.

#### Immunoblotting/Western blot

After the incubation with the compounds described above, the cells were washed with DPBS and treated with full culture medium or starvation medium (Earle’s Balanced Salt Solution, EBSS, Gibco, #24010-043) containing bafilomycin *A*_1_ (Baf *A*_1_, Sigma-Aldrich, #B1793) or a solvent control for 6 h. After the incubation time, the cells were harvested in chilled DPBS by scraping, pelletized at 300 g and 4°C for 5 min and frozen in liquid nitrogen. After cell lysis in lysis buffer (20 mM Tris–HCl, 150 mM NaCl, 500 μM EDTA, 1% [v/v] Triton X-100, 1X protease inhibitor cocktail [Roche, #4693132001] and 1XPhosSTOP [Roche, #04906837001]) for 30 min on ice, the lysates were cleared by centrifugation at 18,000 rcf and 4°C for 15 min and the protein concentration was determined by Bradford assay. Sample buffer was added (62.5 mM Tris, 8.6% [v/v] glycerol, 2% [w/v] SDS, 33.3 μg ml^−1^ bromophenol blue, 1% [v/v] *β*-mercaptoethanol) and the samples were heated at 95°C for 5 min. Equal amounts of protein (25 μg) were subjected to SDS-polyacrylamide gels and afterwards transferred to PVDF membranes (Merck, #IPFL00010). The membranes were blocked with 5% milk powder in TBST and incubated in the indicated primary antibodies (anti-LC3B, CST #2775; p62, PROGEN #gp62-c; Cathespin B, CST #31718; GAPDH, Abcam #ab8245; *β*-Actin, Sigma-Aldrich #A5316) followed by appropriate IRDye 800- or IRDye 680-conjugated secondary antibodies (LI-COR Biosciences #926-68077 #926-32211 #926-32210). The fluorescence signals were detected using an Odyssey Infrared Imaging system (LI-COR Biosciences, Lincoln, NE, USA) and signals were quantified using Image Studio (LI-COR Biosciences, Lincoln, NE, USA). The density of each protein band was divided by the average of the density of all bands of this protein on the membrane. The ratios were normalized to the loading control, and fold changes were calculated by dividing each normalized ratio by the average of the ratios of the control line (full medium). For the quantification of Cathepsin B protein levels in Figure S13, both detected protein bands were included in the quantification.

#### Cathepsin assay

The Cathepsin B and Cathepsin L activity was measured using the Cathepsin Activity Assay Kits (abcam, #ab65300 and #ab65306) according to the manufacturer’s instructions. 10 μg of protein per sample were used in both assays. For inhibitor control, 100 μM Z-FF-FMK (Merck, #219421) was used. The fluorescence of the samples was measured in duplicates with a microplate reader (SynergyMx, BioTek, Winooski, VT, USA) at 505 nm after 405 nm excitation. The average fluorescence intensity of “buffer only” wells without cell lysate was subtracted from the sample fluorescence.

#### Mass spectrometry: Proteomics and secretomics

##### Sample preparation

After incubation with 0.5 mg ml^−1^ CNDs for 48 h (as described above), the cells were carefully washed 6 times with DPBS. Afterwards, the cells were incubated for 6 h in full cell culture medium, basal medium RPMI1640 or in starvation medium EBSS. Only culturing in basal medium or EBSS was selected for secretome analysis, as additives such as FBS would severely impair the detection of secreted proteins. After the incubation, the conditioned medium (basal medium or EBSS) was collected, centrifuged (5 min, 800 g, 4°C) and filtered through a 0.2 μm membrane (Acrodisc, 32 mm Syringe Filter with 0.2 μm Supor Membrane; Pall, # 4652). Aliquots were shock frozen in liquid nitrogen and stored at -80°C. The cells were washed 3 times with chilled DPBS, harvested in DPBS on ice via scraping and pelleted by centrifugation (5 min, 800 g, 4°C). The supernatant was discarded and the cells were stored at -80°C until further processing. A total of 5 replicates for each condition were prepared.

##### Proteomics

Proteins were extracted from frozen cell pellets as described elsewhere.^53^^,^^54^ Briefly, cells were lysed and homogenized in chaotropic lysisbuffer (30 mM Tris, 2 M thiourea, 7 M urea, 4 % CHAPS, pH 8.5; 5 μl per mg cell wet weight) using a TissueLyser (1 min, 40 Hz; Qiagen) and ultrasound (6x 10 s under ice cooling in an ultrasonic bath). After centrifugation (15 min, 16000 rcf, 4°C), supernatants were collected. After determination of protein concentration (Pierce 660 nm Protein Assay, Thermo Fischer Scientific, #22662), samples were adjusted to 0.5 mg ml^−1^ total protein concentration with SDS buffer (final 7.5 % glycerol, 3 % SDS, 37.5 mM Tris/HCl pH 7.0). A quality control was performed by SDS-PAGE using 2 μg total protein per condition and replicate, respectively, and silver staining according to Heukeshoven and Dernick^55^ with slight modifications. 5 μg total protein per condition and replicate were reduced (final 20 mM dithiothreitol, 20 min, 56°C), alkylated (4x molar excess iodoacetamide to dithiothreitol, 15 min, r.t., protected from light), quenched (same amount dithiothreitol as for reduction, 15 min, r.t.) and finally underwent tryptic digestion (200 ng trypsin in 20 μl 50 mM triethylammonium bicarbonate) after applying a slightly modified sp3 protocol^56^ using 50 μg 1:1 mix Sera-Mag SpeedBeads (GE #45152105050250 and #65152105050250) and final 50% ethanol for protein precipitation and 80% ethanol (3x) as well as acetonitrile (1x) for washing the protein-solid phase aggregates. Peptides (25% of input) were reconstituted in 0.1% trifluoracetic acid and subjected to LC-MS analysis.

##### Secretomics

Secretomics was performed as described by Vogt et al.^57^ An aliquot (400 μl) per condition and replicate was thawed on ice in the presence of a protease inhibitor cocktail (added 50 μl of a solution of 1 complete ULTRA tablet, mini, EDTA-free in 2 mL water; Roche, #05892791001), supplemented with SDS buffer (added 50 μl of 30% glycerin, 12% SDS, 150 mM Tris/HCl pH 7.0), reduced (added 40.5 μl of 100 mM dithiothreitol; 20 min at 56°C under shaking), alkylated (added 54 μl of 300 mM iodacetamide; 15 min at r.t. protected from light), and quenched (added 40.5 μl of 100 mM dithiothreitol; 15 min at r.t.). Applying a slightly modified sp3 protocol,^56^ proteins were precipitated (added 10 μl of 20 mg ml^−1^ 1:1 bead-mix of pre-washed Sera-Mag SpeedBeads [GE #45152105050250 and #65152105050250] in water; added 645 μl ethanol abs. p.a.; 15 min at 24°C under shaking), washed (3x 80 % ethanol, 1x acetonitrile) and digested (100 ng trypsin in 20 μl 50 mM triethylammonium bicarbonate). Peptides were reconstituted in 0.1 % trifluoracetic acid and subjected to LC-MS analysis.

##### LC-MS analysis

For the LC-MS analysis, an Orbitrap Fusion Lumos Tribrid mass spectrometer (Thermo Fisher), operated in positive mode and coupled with a nano electrospray ionization source connected with an Ultimate 3000 Rapid Separation liquid chromatography system (Dionex / Thermo Fisher) equipped with an Acclaim PepMap 100 C18 column (75 μm inner diameter, 25 cm length, 2 μm particle size from Thermo Fisher) was applied using a 120 min LC gradient. Capillary temperature was set to 275°C and source voltage to 1.5 kV. MS survey scans had a mass range from 200 to 2000 m/z at a resolution of 120,000. The normalized AGC target was set to 62.5 % and the maximum fill time was 60 ms. A cycle time of 2 s was employed for isolation and fragmentation of the most intensive peptide ions per survey scan by high-energy collision dissociation (HCD).

##### Data analysis

MaxQuant (version 2.1.3.0, Max Planck Institute for Biochemistry, Planegg, Germany) was used for peptide and protein identification and quantification using a human sequence database (UniProtKB, downloaded on 01/27/2021, 75777 entries). Methionine oxidation and N-terminal acetylation were considered as variable modifications and carbamidomethylation at cysteine residues as fixed modification. The identification threshold was set as a false discovery rate of 1 % on protein and peptide level. A total of 3239 protein groups were identified after removing potential contaminants, reverse hits, and proteins only identified by modified peptides (only identified by site).

Both, intensities and label free quantification (LFQ) intensities as measures for relative protein abundance by the MaxQuant software (output proteinGroups.txt file) were statistically analyzed using *R* (version 4.2.0). First, the data were normalized such that the median of the logarithmic (LFQ) intensity differences over all proteins between two samples, respectively, approached zero. As a quality control, a principle component analysis (PCA) using the *prcomp()*-function and cluster analyses using the *heatmap()*-function and cluster methods “ward.D”, “ward.D2”, “single”, “complete”, “average”, “mcquitty”, “median”, and “centroid” were performed. Replicate 1 of the proteome control (no CND treatment) sample using EBSS medium was identified as outlier and omitted in further analyses.

Testing for significant differential protein expression (up- or downregulation; proteomes) or differential protein secretion (secretomes) of the CND treated samples vs. the control samples was performed using the “Significance Analysis of Microarrays” (SAM) analysis method^58^ within the *Siggenes R* package, separately for each of the three (two for secretomes) media. For this approach, a minimum of four valid values had to be present in at least one group (CND treated or control), data were log 2 transformed to reach a normal distribution like data structure, and missing values were filled in with random values from samplewise downshifted normal distributions (0.3 SD width, 1.8 SD downshift). A permutation based false discovery rate (FDR) of 5% was used as significance cutoff. Lysosomal lumenal and lysosomal membrane protein classification was performed according to the list of lysosomal proteins contained in Table S3 of the supplementary material of Richards et al.^59^

### Quantification and statistical analysis

The exact number of replicates measured per experiment is indicated in the corresponding figure legends. Viability assay, cathepsin B/L assay and immunoblot data are represented as mean ± SD. The statistical analysis was performed with OriginPro (Version 2021b, OriginLab Corporation). The statistical tests used are stated in the figure legends. In summary, for the viability assay (Figure 4), cathepsin B/L (Figure 7) and quantification of the lysotracker (Figure 8), one-way ANOVA with Bonferroni comparisons was used. For the immunoblots of CNDs and bPEI-CNDs (Figure 6), two-way ANOVA with Bonferroni comparisons was used. For the immunoblots of bPEI (see supplemental information), three-way ANOVA with Bonferroni comparisons was used. For MS-based proteomics analysis, data analysis with SAM and a FDR ⟨5%  was performed as described above.
