# Supplementary material for: Lysosomal activity in response to the incubation of pristine and functionalized carbon nanodots
Source: iScience. 2024 Dec 25;28(1):111654. doi: 10.1016/j.isci.2024.111654 (PMC11780158; doi:10.1016/j.isci.2024.111654)
Supplement: Document S1. Figures S1–S13 [file mmc1.pdf]

iScience, Volume *28*

Supplemental information

Lysosomal activity in response to the incubation of pristine and functionalized carbon nanodots

Carla Sprengel, Céline David, Lena Berning, Cathrin Nollmann, Thomas Lenz, Kai Stühler, Björn Stork, and Thomas Heinzel

Fig. S1 reproduces the absorption spectra of the CNDs after the different stages of the functionalization. Compared to CNDs, the absorption peak of fCNDs (where the functionalization was only carried out with EDC and NHS but without bPEI in diH_2O) around 350 nm is slightly lowered at the same mass concentration. The fCNDs absorption maxima is 8 nm red shifted to 354 nm. These findings can be explained by the varied mass per particle ratio due to the binding of EDC and NHS as well as modified edge states which may lead to a red shift. The shoulder in the deeper UV region remains around 240 nm but is tilted compared to the pristine CNDs. EDC shows no significant absorption above 250 nm. NHS shows an absorption until 300 nm with a shoulder around 250 nm to 260 nm. Therefore the tilted absorption shoulder of fCNDs around 240 nm can be explained by an overlaying NHS absorption. We note that the fCND particle yield after dialysis (remaining number of particles in the dialysis membrane compared to the initial one) was much lower than the bPEI-CND yield, indicating that the size and mass of fCNDs is lower than the bPEI-CND compounds.

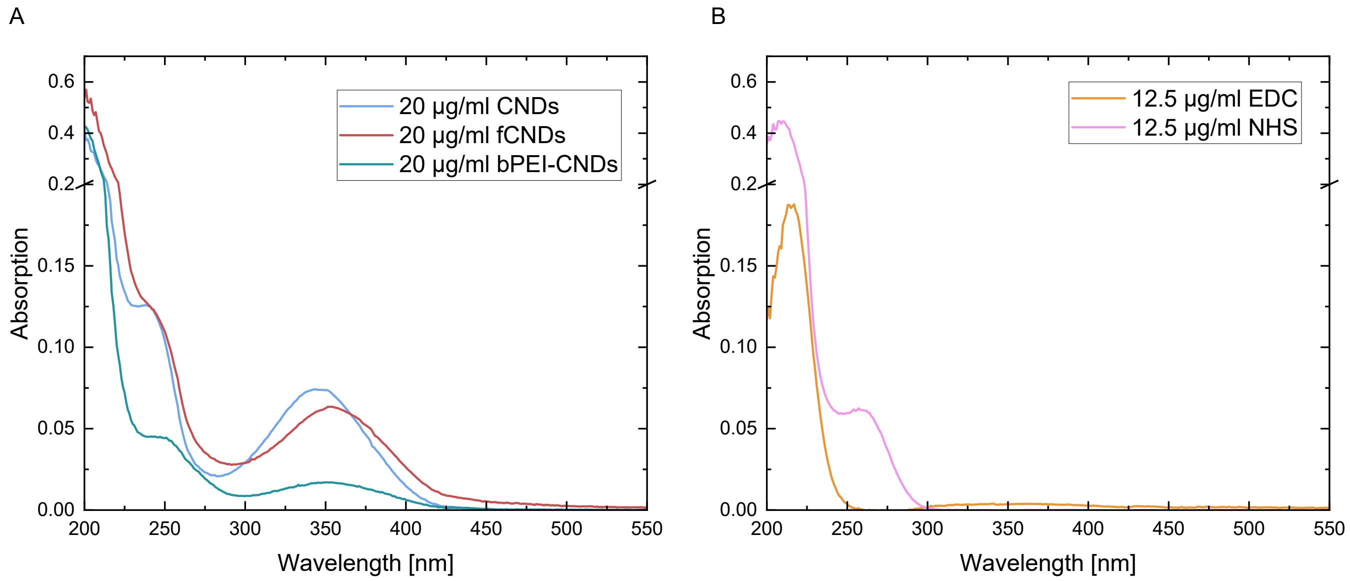

FIG. S1. **Absorption spectra of CNDs, bPEI-CNDs and fCNDs and of NHS and EDC in diH_2O, related to Figure 1.**

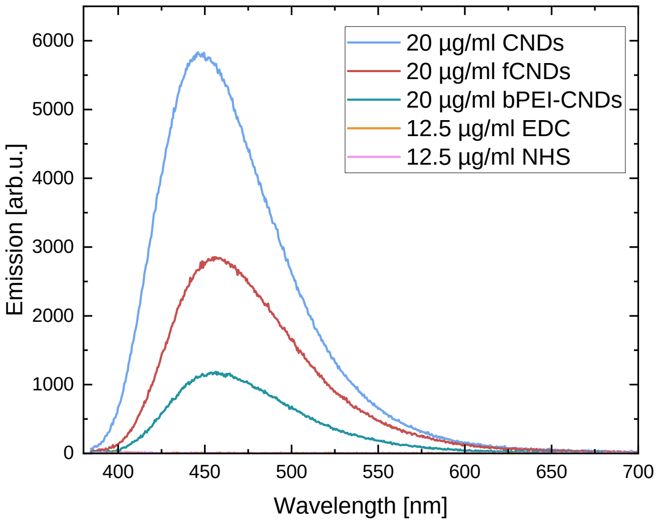

FIG. S2. **Emission spectra of CNDs, fCNDs, bPEI-CNDs, NHS and EDC after excitation with 360 nm, related to Figure 1.**

The emission spectra of CNDs, fCNDs, bPEI-CNDs, NHS and EDC after excitation with 360 nm are shown in Fig. S2. The fCNDs show a red shift of the emission maximum to 456 nm. The emission intensity is lowered by approximatly a factor of 2. This can be explained by the different edge groups of the CNDs which may cause nonradiative relaxations. NHS and EDC show no fluorescence emission after excitation with 360 nm.

In Fig. S3, S4 and S5, we show the ^{1}H-NMR spectrum of the pristine CNDs, of the CNDs functionalized with bPEI and for reference purposes of free bPEI, respectively. As described in the main text, the coupling between the CNDs and the bPEI leads to a superposition of the spectra of the components, accompanied by a marked broadening of the resonances.

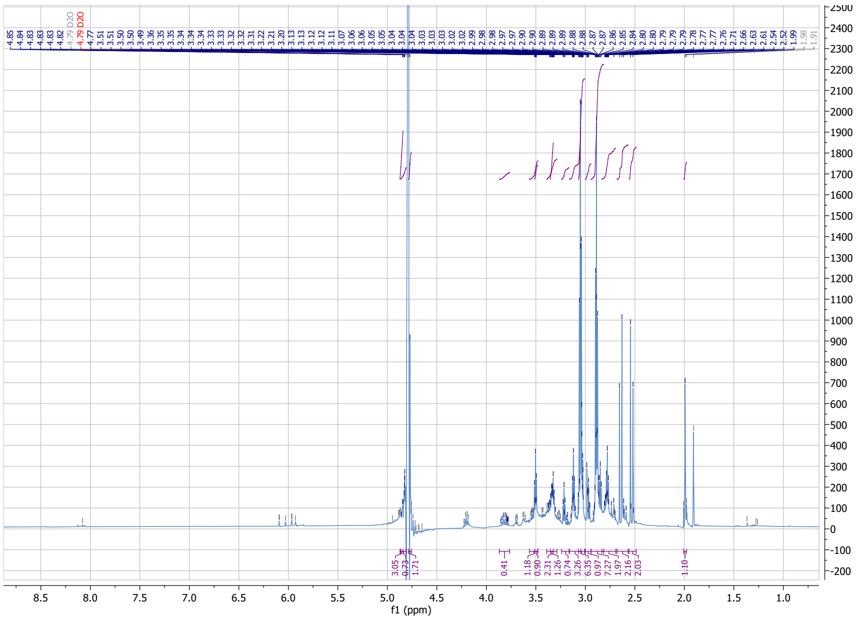

FIG. S3. **^{1}H-NMR spectrum of the CNDs, related to Figure 2.**
Single peaks are marked in blue and multiplets in purple. The chemical shifts amount to: ^{1}H-NMR (600 MHz, D_2O) δ[ppm]= 4.86 (s, 3H), 4.86 – 4.81 (m, 1H), 4.77 (s, 2H), 3.87 – 3.76 (m, 0H), 3.56 – 3.48 (m, 1H), 3.50 (s, 1H), 3.34 (s, 2H), 3.39 – 3.29 (m, 1H), 3.24 – 3.16 (m, 1H), 3.16 – 3.05 (m, 3H), 3.07 – 3.01 (m, 6H), 3.00 – 2.94 (m, 1H), 2.94 – 2.81 (m, 7H), 2.83 – 2.69 (m, 2H), 2.64 (d, J = 15.0 Hz, 2H), 2.53 (d, J = 15.0 Hz, 2H), 1.99 (s, 1H)

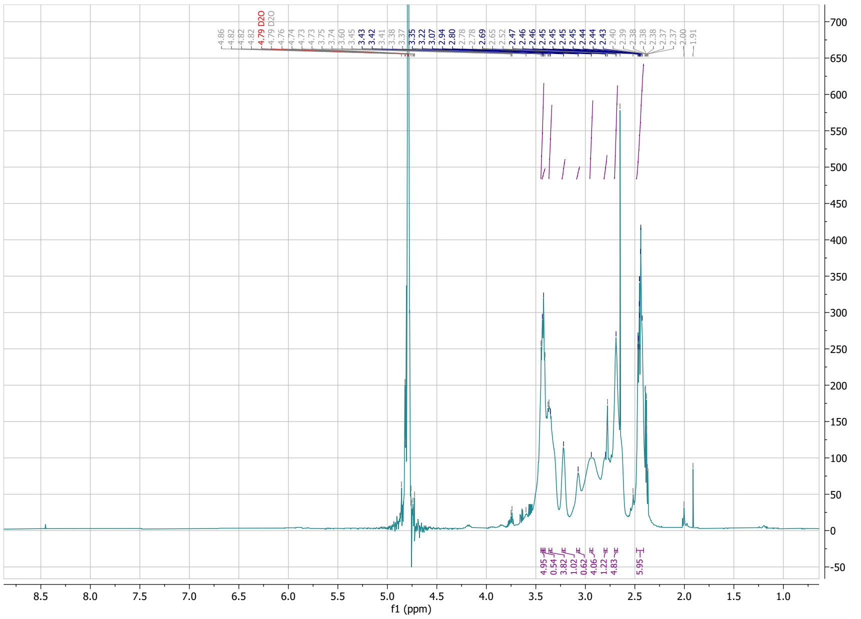

FIG. S4. [1]**H-NMR spectrum of the bPEI-CNDs, related to Figure 2.**
Single peaks are marked in blue and multiplets in purple. The chemical shifts amount to: ^{1}H-NMR (600 MHz, D_2O) δ[ppm]= 3.43 (s, 5H), 3.42 (s, 1H), 3.35 (s, 4H), 3.22 (s, 1H), 3.07 (s, 1H), 2.94 (s, 4H), 2.80 (s, 1H), 2.69 (s, 5H), 2.48 – 2.41 (m, 6H)

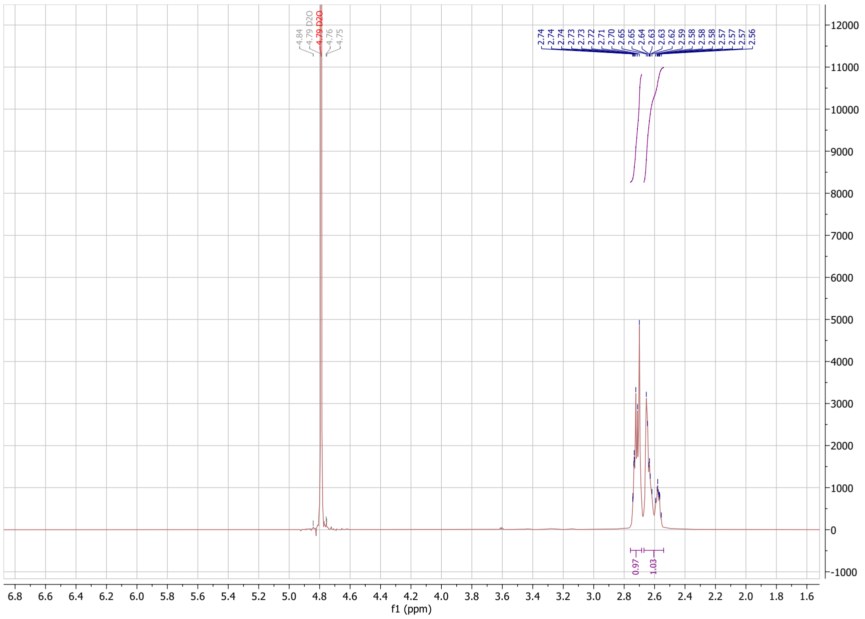

FIG. S5. [1]**H NMR spectrum of free bPEI, related to Figure 2.**
Single peaks are marked in blue and multiplets in purple. The chemical shifts amount to: ^{1}H-NMR (600 MHz, D_2O) δ[ppm]= 2.72 (dd, J = 13.5, 7.0 Hz, 1H), 2.67 – 2.54 (m, 1H)

Fig. S6 illustrates the spatial correlation between the fluorescence of the lysotracker and the CNDs. They form the basis of our cross correlation analysis given in the main text. Note in particular the significantly decreased CND fluorescence background in the bPEI cells (B) as compared to the cells incubated with pristine CNDs (A), indicating an enhanced localization in the lysosomes.

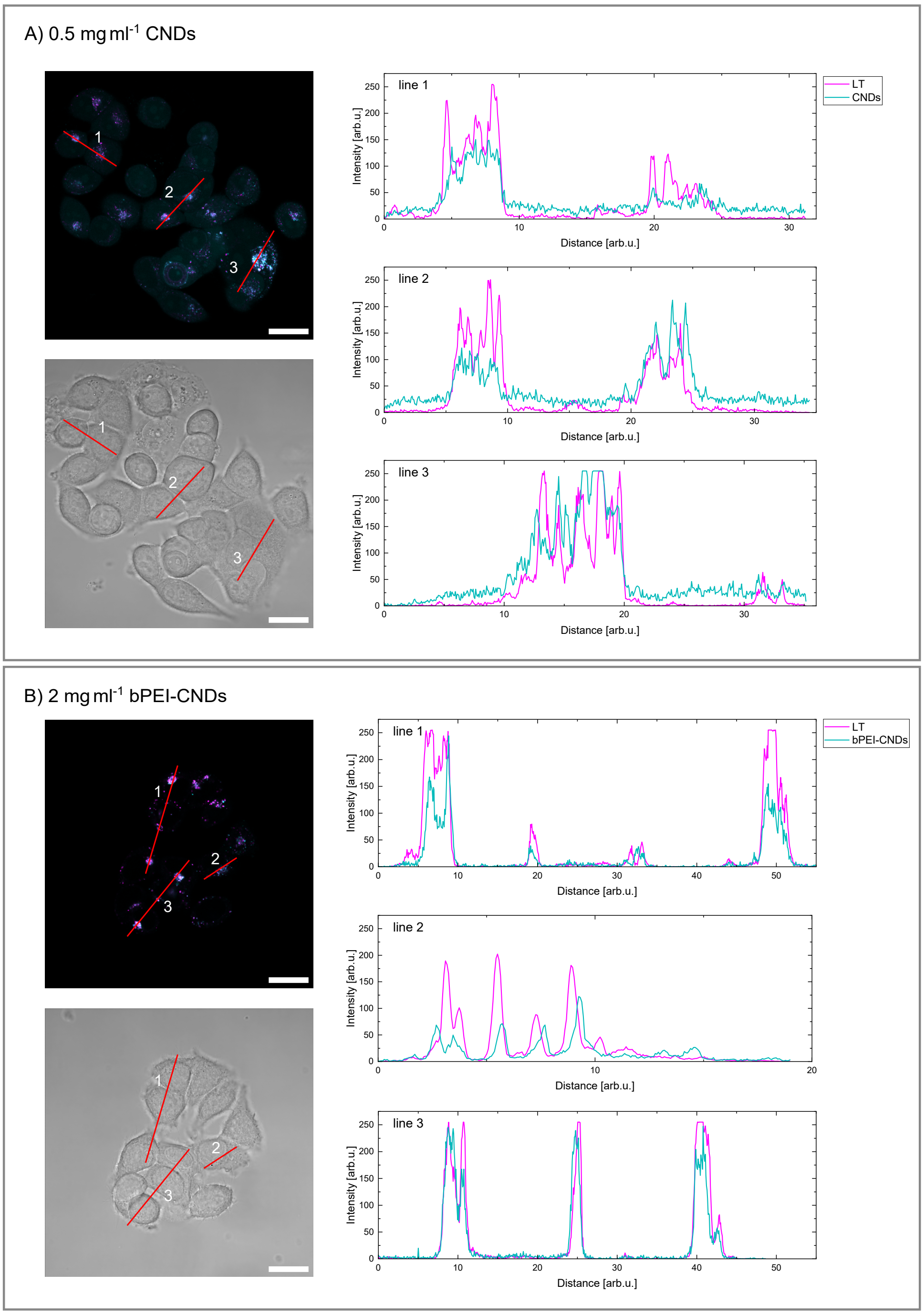

FIG. S6. **Representative line plots of the microscopy images, related to Figure 3.**
A: MCF-7 cells incubated with $0.5\,\mathrm{mg\,ml^{-1}}$ CNDs. Shown are the overlay of the magenta lysotracker ("LT") channel and the cyan (bPEI)-CND channel, the transmitted light image as well as three line plots per sample displaying the fluorescence intensity of the (bPEI)-CNDs in cyan and lysotracker in magenta. The lines used for the intensity line plots are depicted in red and labeled in the corresponding microscopy images.
B: As in A, but for MCF-7 cells incubated with $2\,\mathrm{mg\,ml^{-1}}$ bPEI-CNDs.

Additional microscopy images to the one shown in the main text after incubation with bPEI-CNDs and free bPEI are illustrated in Fig. S7.

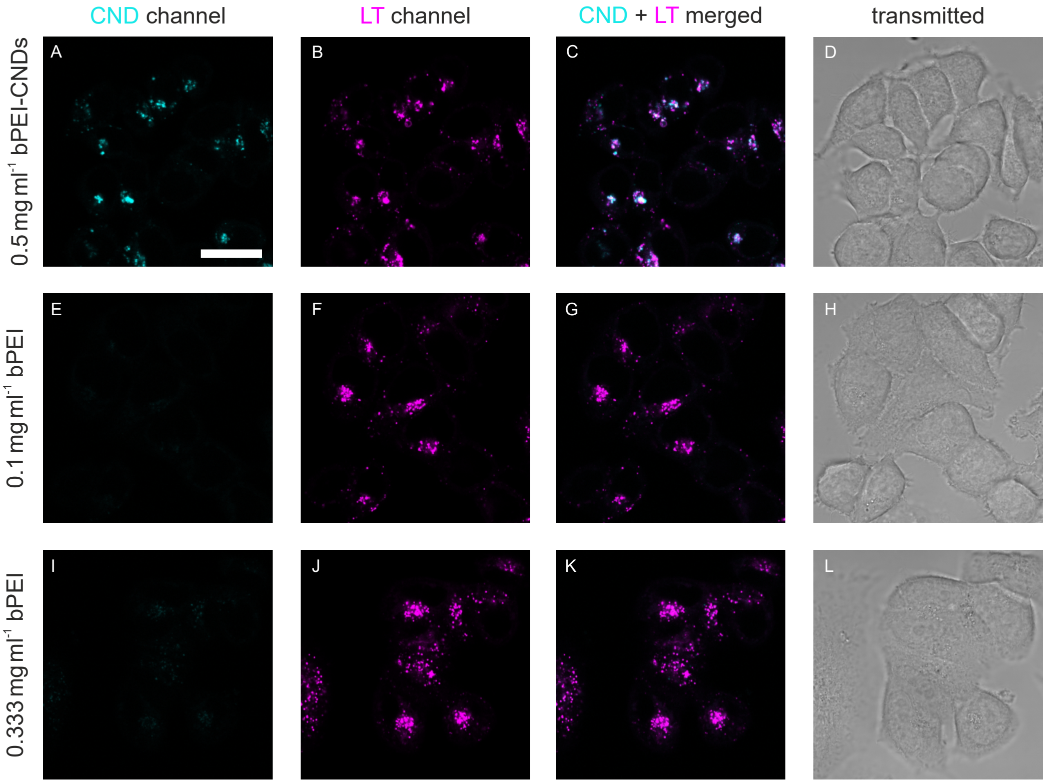

FIG. S7. **Confocal microscopy images of MCF-7 cells incubated with bPEI-CNDs or bPEI, related to Figure 3.** A-D: MCF-7 cells incubated with 0.5 $\mathrm{mg\,ml^{-1}}$ bPEI-CNDs. Lysosomes are stained with lysotracker. The CND channel is shown in cyan, the lysotracker ("LT") channel in magenta. The scale bar of 20 µm shown in A applies to all images.
E-H: MCF-7 cells incubated with 0.1 $\mathrm{mg\,ml^{-1}}$ bPEI.
I-L: MCF-7 cells incubated with 0.333 $\mathrm{mg\,ml^{-1}}$ bPEI.

In Fig. S8, the viability measurements 72 h and 96 h after the incubation are shown. The inertness of the CNDs still holds over these periods, while the damage due to the bPEI linked to the CNDs, becomes significant after 96 hours.

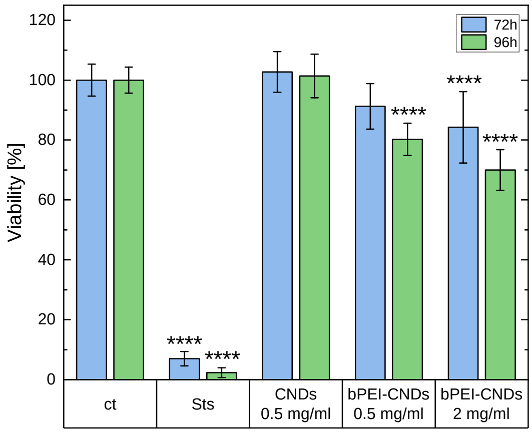

FIG. S8. **Viability of MCF-7 cells after incubation for 72 h and 96 h with CNDs and bPEI-CNDs, related to Figure 4.**
Staurosporine (Sts) was used as a positive control. Data are represented as mean ± SD of N=4 biological replicates with each n=3 technical replicates. p values were determined by two-way ANOVA with Bonferroni comparison. In comparison with the control sample "ct" with the same incubation time: ****$p<0.0001$

Fig. S9 gives the ImageJ macro code used to process and analyze the microscopy images.

```
close("*");
//programm written for .czi images. Images should all be obtained with same measurement parameters.

//define directorys
inputdir = getDirectory("input");
dir = getDirectory("output");
dir2  = getDirectory("Zell-ROIs"); //in this directory, cell ROIs (each cell is one ROI) should be saved in zip files with the name: [title of image].czi_cells.zip
dir3  = getDirectory("Lysosome areas");

list = getFileList(inputdir);
for (p = 0; p < list.length; p++) {

//open image from the input directory and get infos
open(inputdir+list[p]);
title = File.nameWithoutExtension;
title2 = getInfo("image.filename");

imagedir = getInfo("image.directory");

//start ROI manager and open the previously defined cell ROIs
run("ROI Manager...");
roiManager("Open", dir2+title+".czi_cells.zip");

n = getNumber("How many cell-ROIs are shown in the ROI-Manager?", 10);


//start image analysis
for (i = 0; i < n; i++) {
//use a duplicate for analysis to not overwrite the original image:
selectWindow(title2);
run("Duplicate...", "duplicate");
//Name of the duplicate will be Name-1.czi

selectWindow(title+"-1.czi");

//Start image processing with convoluted background subtraction and intensity threshold setting. Make image binary for further process. Intensity threshold should be set once for all images.
Stack.setPosition(1,1,1);
run("Convoluted Background Subtraction", "convolution=Median radius=10 slice");
setThreshold(10, 255);
run("Make Binary", "calculate only black");
run("Watershed", "slice");

//ROI manager will be opened to start with the analysis of each cell separately
run("ROI Manager...");
roiManager("Show All");
roiManager("Delete");
roiManager("Show None");
roiManager("Open", dir2+title+".czi_cells.zip");

x=i+1;

selectWindow("ROI Manager");
roiManager("Select",i);
//lysosome channel should be the first image in the stack.
selectWindow(title+"-1.czi");
Stack.setPosition(1,1,1);

//use particle analyzer to count particles and determine size. Results should be saved (e.g. in excel or txt table) an can be further analysed
run("Analyze Particles...", "size=0.01-Infinity show=Outlines exclude clear summarize add slice");

//sav an image of the lysosomes of each cell
Stack.setPosition(1,1,1);
saveAs("Tiff", dir+title+"_Bild_lysosomen"+"_cell"+x);
close();


selectWindow(title+"-1.czi");
close();

//save lysosome ROIs
selectWindow("ROI Manager");
roiManager("Save", dir+title+"lysosomes_cell"+x+"_ROI.zip");
run("Close");

//close all open windows for analysis of next image
selectWindow(title2);
close("\\Others");

run("ROI Manager...");
roiManager("Open", dir2+title+".czi_cells.zip");

}
run("ROI Manager...");
roiManager("Show All");
roiManager("Delete");
roiManager("Show None");
}
```

FIG. S9. **ImageJ Macro for lysosomal analysis, related to Figure 8 and STAR Methods.**

Fig. S10 reproduces the volcano plots for both the proteome (top) and the secretome (bottom) studies. The analysis of differential protein abundances after the incubation of MCF-7 cells with CNDs was performed in different media, namely full medium "RPMI", basal medium RPMI1640 "RPMI-" and EBSS as indicated.

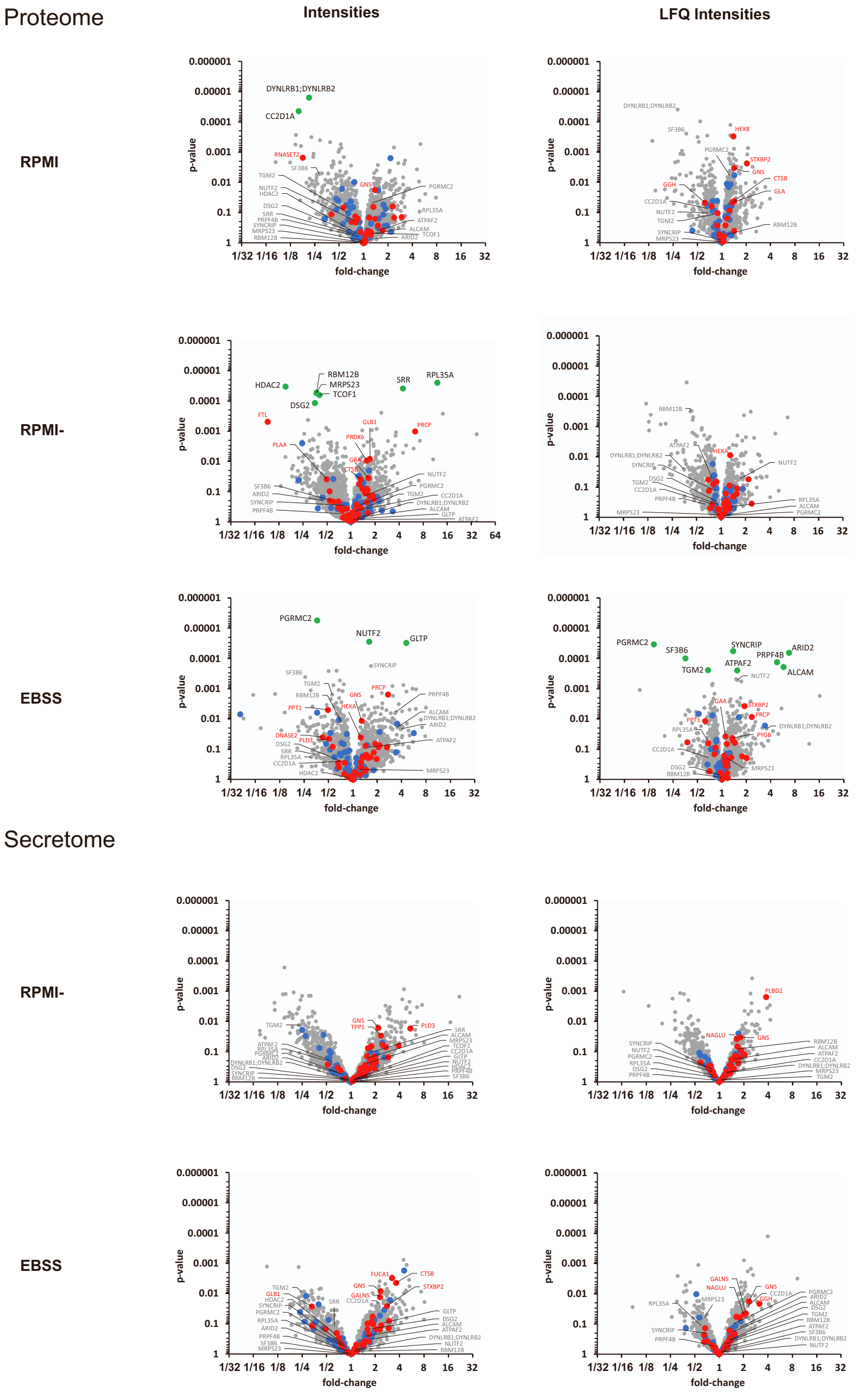

FIG. S10. **Volcano plots based on intensity or LFQ intensity values (MaxQuant) for MS-based proteomics (top) and secretomics (bottom), related to Figure 5.** Proteins with a permutation-based FDR (SAM analysis) $< 5\,\%$ are indicated by green data points and labelled with their gene names in black for the respective conditions or in grey, in case this criterion was met for other conditions. Lysosomal lumenal and lysosomal membrane proteins are labelled with blue and red data points, respectively, and lumenal proteins with p-value < 0.05 for the respective conditions are labelled with red gene names.

The results of the a functional enrichment analysis using the STRING database [1] for the significantly altered proteins from the proteome analysis is shown in Fig. S11. We used the following analysis parameters: String Version 12.0, minimum required interaction score: 0.4. Network Stats: number of nodes: 19, number of edges: 1, average node degree: 0.105, avg. local clustering coefficient: 0.105, expected number of edges: 2, PPI enrichment p-value: 0.887.

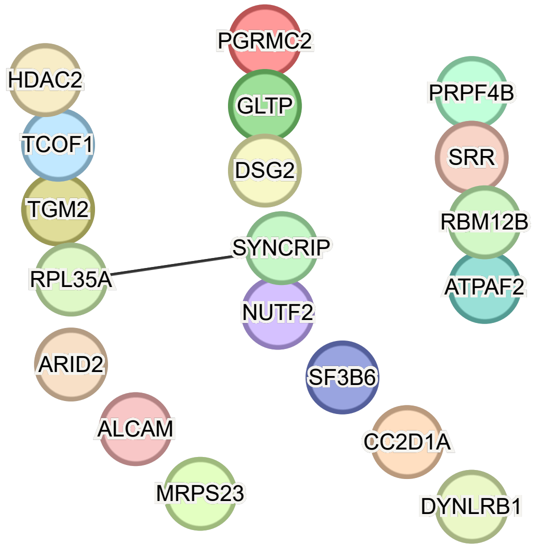

FIG. S11. **Results of the functional enrichment analysis using the STRING database, related to Figure 5 and S10.** The black line between *RPL35A* and *SYNCRIP* indicates co-expression of those two proteins.

Fig. S12 gives an overview of the levels of p62 and LC3 in cells that have been exposed to bPEI in different media.

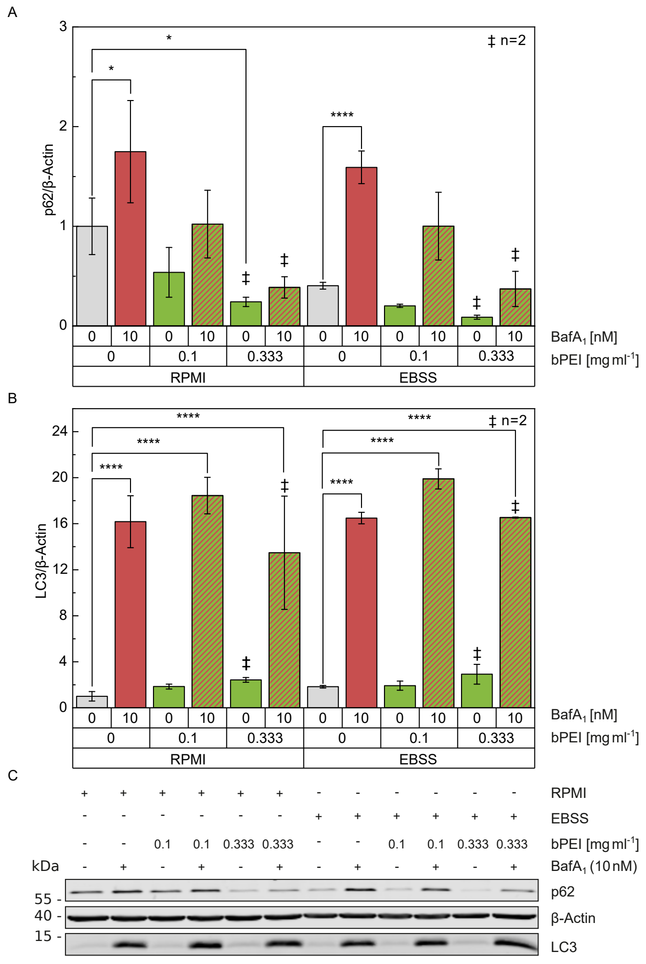

FIG. S12. **Analysis of p62 and LC3 levels after incubation with bPEI, related to Figure 6.**
A,B: Quantification of (A) p62 levels und (B) LC3 levels of cells that are exposed to bPEI for 48 h and/or $BafA_1$ in full medium "RPMI" or starvation medium "EBSS" for 6 h. The levels were quantified and normalized to β-Actin. Data are represented as mean ± SD of N=3 biological replicates (N=2 for 0.333 mg ml^{-1} bPEI-Samples). p values were determined by three-way ANOVA with Bonferroni comparison. Only the significance values compared to the control samples "ct" in the same medium are shown. *p<0.05, ****p<0.0001.
C: A representativ immunoblot is shown.

The corresponding picture for the Cathepsin B levels is given in Fig. S13.

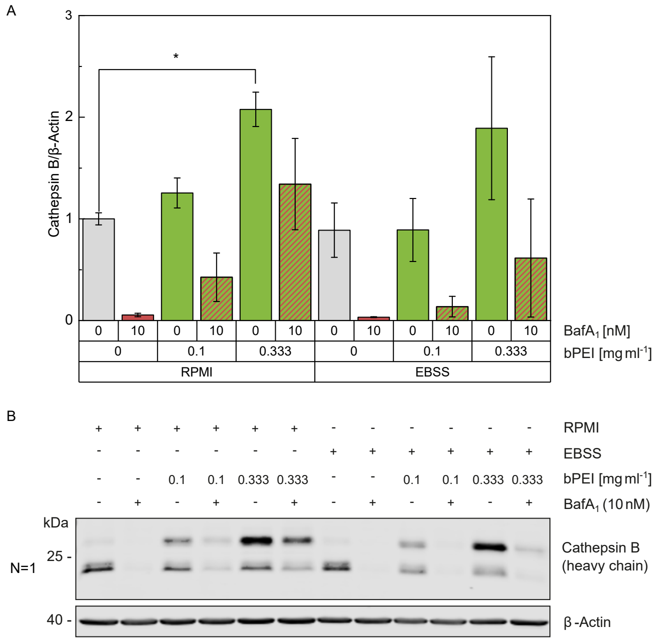

FIG. S13. **Analysis of cathepsin B levels after incubation with bPEI, related to Figure 7.**
A: Cathepsin B levels of cells that are exposed to bPEI for 48 h and/or $BafA_1$ in full medium "RPMI" or starvation medium "EBSS" for 6 h. The levels were quantified and normalized to β-Actin. Data are represented as mean ± SD of N=2 biological replicates. p values were determined by three-way ANOVA with Bonferroni comparison. Only the significance values compared to the control samples "ct" in the same medium are shown. *$p<0.05$.
B: A representativ immunoblot is shown.

[1] D. Szklarczyk, R. Kirsch, M. Koutrouli, K. Nastou, F. Mehryary, R. Hachilif, A. L. Gable, T. Fang, N. T. Doncheva, S. Pyysalo, P. Bork, L. J. Jensen, and C. von Mering, The string database in 2023: protein–protein association networks and functional enrichment analyses for any sequenced genome of interest, Nucleic Acids Research **51**, D638 (2023).
